# Supplementary material for: Broad-spectrum activity of bulevirtide against clinical isolates of HDV and recombinant pan-genotypic combinations of HBV/HDV
Source: JHEP Rep. 2023 Aug 22;5(11):100893. doi: 10.1016/j.jhepr.2023.100893 (PMC10622701; doi:10.1016/j.jhepr.2023.100893)
Supplement: Multimedia component 1 [file mmc1.pdf]

# Broad-spectrum activity of bulevirtide against clinical isolates of HDV and recombinant pan-genotypic combinations of HBV/HDV

Roberto Mateo, Simin Xu, Alex Shornikov, Tahmineh Yazdi, Yang Liu, Lindsey May, Bin Han, Dong Han, Ross Martin, Savrina Manhas, Christopher Richards, Caleb Marceau, Thomas Aeschbacher, Silvia Chang, Dmitry Manuilov, Julius Hollnberger, Stephan Urban, Tarik Asselah, Dzhamal Abdurakhmanov, Pietro Lampertico, Evguenia Maiorova, Hongmei Mo

Table of contents

Table S1....................................................................................................2

Table S2....................................................................................................3

Table S3....................................................................................................4

Table S4....................................................................................................6

## Table S1. Percentage of HDAg-positive PHH cells after infection with HDV-1-8 enveloped with L-HBsAg from HBV GTA-H

| | BLV $EC_{50}$ (nM) | | | | | | | | | |
|---|---|---|---|---|---|---|---|---|---|---|
| HBV GT | HDV-1 | HDV-2 | HDV-3 | HDV-4 | HDV-5 | HDV-6 | HDV-7 | HDV-8 | HBV mean | HBV median |
| GTA | 0.21 ± 0.03 | 0.17 ± 0.04 | 0.66 ± 0.33 | 0# | 2.55 ± 0.07 | 4.19 ± 0.30 | 0.95 ± 0.38 | 3.75 ± 0.07 | 1.56 | 0.81 |
| GTB | 18 ± 5.63 | 8.27 ± 0.29 | 10.77 ± 3.60 | 2.27 ± 0.48 | 6.91 ± 0.21 | 9.84 ± 0.44 | 5.54 ± 0.25 | 4.38 ± 0.06 | 8.36 | 7.59 |
| GTC | 0.77 ± 0.14 | 0.39 ± 0.07 | 0.08 ± 0.04 | 0.02 ± 0.01 | 2.55 ± 0.28 | 0.54 ± 0.17 | 0.21 ± 0.04 | 3.20 ± 0.09 | 0.97 | 0.47 |
| GTD | 10.1 ± 5.54 | 0.53 ± 0.03 | 12.40 ± 2.27 | 0.05 ± 0.0 | 1.45 ± 0.27 | 2.07 ± 0.35 | 0.30 ± 0.03 | 1.94 ± 0.10 | 3.60 | 1.70 |
| GTE | 2.76 ± 0.27 | 6.60 ± 0.57 | 6.96 ± 1.60 | 0.47 ± 0.01 | 34..42[a] | 24.77[a] | 25.14 ± 3.68 | 11.36 ± 1.25 | 14.06 | 9.16 |
| GTF | 1.19 ± 0.43 | 0.02 ± 0.01 | 1.96 ± 0.33 | 0 | 0.38 ± 0.16 | 0 | 0.08 ± 0.02 | 4.09 ± 0.23 | 1.29 | 0.79 |
| GTG | 13.3 ± 4.68 | 0.86 ± 0.11 | 6.40 ± 0.41 | 0.36 ± 0.14 | 24.94[a] | 3.88 ± 1.16 | 12.85 ± 4.53 | 4.00 ± 0.08 | 8.32 | 5.20 |
| GTH | 1.57 ± 0.47 | 0.08 ± 0.04 | 5.40 ± 0.40 | 0 | 0.52 ± 0.13 | 0 | 0.03 ± 0.01 | 3.53 ± 0.03 | 1.39 | 0.30 |
| HDV mean | 6.09 | 2.12 | 5.58 | 0.45 | 9.21 | 6.47 | 5.64 | 4.53 | | |
| HDV median | 2.16 | 0.46 | 5.90 | 0.05 | 2.55 | 3.88 | 0.63 | 3.88 | | |

[a] Assay used 40 µl of input viral inoculum (N=1)

## Table S2. Activity of BLV against HDV genotype 1 enveloped with representative sequences of preS1 region for HBV genotypes A-H

| Position[a] | Average $EC_{50}$ (nM)[c] | 15 20 25 30 35 40 45 50 55 |
|---|---|---|
| **BLV** | - | G T N L S V P N P L G F F P D H Q L D P A F G A N S N N P D W D F N P N K D H W P E A N K V G |
| **GTA pLX304-HB2.7-gtA** | **0.65** | . . . . . . . . . . . . . . . . . . . . V . . . . . . . . . . . . . . I . . . . . A . . Q . . |
| **GTB pLX304-HB2.7-gtB** | **0.90** | . . . . . . . . . . . . . . . . . . . . . . K . . . E . . . . . L . . Y . . N . . D . . . . . |
| **GTC pLX304-HB2.7-gtC** | **0.46** | . . . . . . . . . . . . . . . . . . . . . . . . . . . . . . . . . . . . . . . . . . . . Q . . |
| **GTD pLX304-HB2.7-gtD** | **0.49** | . Q . . . T S . . . . . . . . . . . . . . . R . . T A . . . . . . . . . . . T . . D . . . . . |
| **GTE pLX304-HB2.7-gtE** | **0.85** | . K . H . T T . . . . . . . . . . . . . . . R . . T R . . . . . H . . . . . . . T . . . . . . |
| **GTF pLX304-HB2.7-gtF** | **0.28** | . Q . . . . . . . . . . . . . . . . . . L . R . . . S S . . . . . . T . . . S . . M . . . . . |
| **GTG pLX304-HB2.7-gtG** | **0.57** | . K . . . T S . . . . . L . . . . . . . . . R . . T . . . . . . . . . K . . P . . . . . . . . |
| **GTH pLX304-HB2.7-gtH** | **0.45** | . Q . . . . . . . . . . . . . . . . . . L . R . . . S S . . . . . . T . . . N . . M . . . . . |

[a] Numbering corresponds to positions of genotype A X02763
[b] BLV sequence was used as a reference for each position
[c] Average of two replicate values is shown (N=2)

## Table S3. Sequence alignment of BLV sequence with the most common clinical isolates of HBV genotypes A-D

| Position | Average EC50 (nM)[a] | 15 20 25 30 35 40 45 50 55 |
|---|---|---|
| BLV Sequence | - | GTN LSVPN PLGFF PDHQL DPAFG ANSNN PDWDF NPNKD HWPEA NKVG |
| GTA Consensus | 0.65[b] | ... ..... ..... ..... ..... ..... ..... ..I.. ...A. .Q.. |
| HBVA.1-HDV1 | 0.17 | ... .A... ..... ..... .L..R ..... ..... ..V.. -..Q. .Q.. |
| HBVA.2-HDV1 | 0.88 | ... ..... ..... ..... ..... ..... ..... ..I.. ...Q. .Q.. |
| GTB Consensus | 0.90[b] | ... ..... ..... ..... ....K ...E. ....L ..H.. N..D. .... |
| HBVB.1-HDV1 | 0.47 | ... .A... ..... ..... ..L.R ...E. ....L ..H.. N..D. .... |
| HBVB.2-HDV1 | 0.74 | ... ..... ....L ..... ....K ...D. ....L ..H.. T..DS .... |
| HBVB.3-HDV1 | 0.55 | ... ..... ....L ..... ....K ...D. ....L ..H.. N..D. .... |
| HBVB.4-HDV1 | 0.42 | ... ..... ..... ..... ....K ...D. ....L ..H.. Y..DS .... |
| HBVB.5-HDV1 | 0.41 | ... ..... ..... ..... ....K ...E. ..... .QH.. N..D. .... |
| HBVB.6-HDV1 | 0.93 | ... ..... ..... ..... ....K ...E. ....L ..Y.. N..D. .... |
| HBVB.7-HDV1 | 0.67 | ... ..... ..... ..... ....K ...E. ....L ..Y.. N..D. .... |
| HBVB.8-HDV1 | n/a | ... ..... ..... ..... ....K ...E. ....L ..H.. T..D. .... |
| HBVB.9-HDV1 | 0.68 | ... ..... ..... ..... ....K ...E. ....L ..Y.. N..D. .... |
| HBVB.10-HDV1 | 0.43 | ... ..... ..... ..... ....K ...E. ....L ..H.. N..N. .... |
| GTC Consensus | 0.46[a] | ... ..... ..... ..... ..... ..... ..... ..... ..... .Q.. |
| HBVC.1-HDV1 | 0.47 | ... .A... ..... ..... .L..R ..... ..... ..... P.... .Q.. |
| HBVC.2-HDV1 | 0.21 | ... .A... ..... ..... .L..R ..... ..... ..... P.... HQ.. |
| HBVC.3-HDV1 | 0.71 | ... ..... ....L ..... ..... ..... ..... ..... ..... .Q.. |
| HBVC.4-HDV1 | 0.24 | ... .A... ..... ..... .L..R ..... ..... ..... P.... .Q.. |
| HBVC.5-HDV1 | 0.45 | ... ..... ..... .G... ....R ..... ..... ..... Q..A. .Q.. |
| HBVC.6-HDV1 | 0.28 | ... ..... ..... .G... ..... ..... ....L ..... Q..A. HQ.. |
| HBVC.7-HDV1 | 0.88 | ... ..... ....L ..... ..... ..... ..... ..... P.... WQ.. |
| HBVC.8-HDV1 | 0.29 | ... ..... ..... .G... ..... ..... ..... ..... Q..A. .Q.. |
| HBVC.9-HDV1 | n/a | ... ..... ..... ..... ..... ..... ..... ..... Q.... .Q.. |

| | | |
|---|---|---|
| **HBVC.10-HDV1** | 0.26 | . . . . . . . . . . . . . . G . . . . . . . . . . . . . . . . . . . . . . . . . Q . . A . . Q . . |
| **GTD Consensus** | 0.49[a] | . Q . . . T S . . . . . . . . . . . . . . . R . . T A . . . . . . . . . . . T . . D . . . . . |
| **HBVD.1-HDV1** | 0.29 | . Q . . . T S . . . . . . . . . . . . . . . K . . T A . . . . . . . . . . . P . . D . . . . . |
| **HBVD.2-HDV1** | 0.2 | . Q . . . T S . . . . . . . . . . . . . . . R . . T A . . . . . . . . . . . T . . N . . . . . |

[a] Average of two replicate values is shown (N=2)
[b] $EC_{50}$ for representative sequences for HBV genotypes A-D were measured as described in Table 1
n/a: $EC_{50}$ values not available

**Table S4. Subject clinical, virological and biochemical information for baseline plasma samples from participants described in this study**

| Subject ID | Country[a] | HBV GT | HDV GT | Log10 HDV RNA [IU/mL] | HBV DNA [IU/mL] | Log10 HBsAg [IU/mL] | $EC_{50}$ | ALT [U/mL] |
|---|---|---|---|---|---|---|---|---|
| 1 | RUS | D | 1 | 4.81 | <10 | 3.18 | 0.37 | ND |
| 2 | RUS | D | 1 | 4.74 | <10 | 3.52 | 0.22 | ND |
| 3 | RUS | D | 1 | 7.01 | 22 | 3.08 | 0.69 | ND |
| 4 | RUS | D | 1 | 5.21 | 15 | 3.92 | 0.26 | ND |
| 5 | RUS | D | 1 | 6.53 | 26 | 3.88 | 0.25 | ND |
| 6 | RUS | D | 1 | 6.64 | 10 | 4.08 | 0.16 | ND |
| 7 | RUS | A | 1 | 6.52 | 522 | 3.73 | 0.57 | ND |
| 8 | RUS | D | 1 | 7.01 | 60 | 4.23 | 0.68 | ND |
| 9 | RUS | D | 1 | 6.21 | 22 | 3.40 | 0.19 | ND |
| 10 | RUS | D | 1 | 4.01 | <10 | 2.85 | 0.23 | 439 |
| 11 | FRA | A | 1 | 5.23 | 20 | 3.96 | 0.77 | ND |
| 12 | FRA | E | 5 | 5.85 | 768 | 4.18 | 0.35 | ND |
| 13 | FRA | A | 6 | 4.13 | 16 | 3.45 | 0.70 | 59 |
| 14 | FRA | E | 5 | 6.37 | ND | 3.79 | 0.72 | 76 |
| 15 | RUS | D | 1 | 3.75 | 14 | 3.91 | 0.40 | 81 |
| 16 | RUS | D | 1 | 4.49 | 71 | 4.00 | 0.41 | 113 |
| 17 | RUS | D | 1 | 5.04 | 19 | 3.76 | 0.24 | 302 |
| 18 | RUS | D | 1 | 6.55 | 13 | 4.48 | 0.27 | 104 |
| 19 | RUS | D | 1 | 7.46 | 11 | 4.15 | 1.27 | 94 |
| 20 | RUS | D | 1 | 5.69 | 12 | 3.76 | 0.67 | 42 |
| 21 | RUS | D | 1 | 4.56 | 12 | 3.83 | 0.47 | 45 |
| 22 | RUS | D | 1 | 7.06 | 172 | 4.18 | 0.88 | 70 |
| 23 | RUS | D | 1 | 4.92 | <10 | 2.49 | 0.29 | 335 |
| 24 | RUS | D | 1 | 4.15 | <10 | 3.91 | 0.47 | 49 |
| 25 | RUS | D | 1 | 6.52 | 28 | 4.23 | 0.27 | 95 |
| 26 | RUS | D | 1 | 3.78 | 66 | 3.76 | 0.24 | ND |
| 27 | RUS | D | 1 | 6.03 | 93 | ND | 0.87 | ND |
| 28 | RUS | D | 1 | 4.83 | 21 | 3.77 | 0.50 | 82 |
| 29 | RUS | D | 1 | 6.49 | 16 | 2.70 | 0.31 | 133 |
| 30 | RUS | D | 1 | 4.83 | 10 | 3.66 | 0.43 | 36 |
| 31 | RUS | D | 1 | 5.56 | 185 | 3.32 | 0.42 | 82 |
| 32 | RUS | D | 1 | 4.97 | 43333 | 3.90 | 0.39 | 193 |
| 33 | RUS | D | 1 | 4.61 | <10 | 3.75 | 0.51 | 91 |
| 34 | RUS | D | 1 | 7.49 | 22 | 4.30 | 0.43 | 285 |
| 35 | RUS | D | 1 | 4.79 | 12 | 3.95 | 0.32 | 40 |
| 36 | RUS | D | 1 | 3.86 | 10 | 2.54 | 0.34 | 64 |
| 37 | RUS | D | 1 | 5.27 | <10 | 3.57 | 0.80 | 39 |
| 38 | RUS | D | 1 | 3.90 | 57 | 3.69 | 0.22 | 212 |
| 39 | RUS | D | 1 | 5.58 | 3318 | 4.46 | 0.32 | 123 |

| | | | | | | | | |
|---|---|---|---|---|---|---|---|---|
| 40 | RUS | D | 1 | 5.50 | 39 | 4.18 | 0.26 | 145 |
| 41 | RUS | D | 1 | 6.05 | 148 | 4.43 | 0.28 | 273 |
| 42 | RUS | D | 1 | 3.84 | 11 | 3.04 | 0.49 | 273 |
| 43 | RUS | D | 1 | 5.40 | <10 | 4.11 | 0.20 | 107 |
| 44 | RUS | D | 1 | 4.01 | <10 | 4.11 | 0.42 | ND |
| 45 | RUS | D | 1 | 5.38 | 317 | 3.36 | 0.39 | ND |
| 46 | RUS | D | 1 | 5.28 | 13 | 3.30 | 0.43 | 73 |
| 47 | RUS | D | 1 | 5.16 | <10 | 3.94 | 0.25 | 125 |
| 48 | RUS | D | 1 | 6.08 | 418 | 4.28 | 0.35 | 54 |
| 49 | RUS | D | 1 | 7.04 | 3343101 | 3.99 | 1.27 | 210 |
| 50 | RUS | D | 1 | 4.39 | 26 | 3.58 | 0.30 | 38 |
| 51 | RUS | D | 1 | 6.06 | 125 | 4.15 | 0.31 | 141 |
| 52 | RUS | D | 1 | 6.12 | 101 | 4.20 | 0.23 | 101 |
| 53 | RUS | D | 1 | 5.63 | 17 | 3.91 | 0.28 | 97 |
| 54 | RUS | D | 1 | 6.19 | 152 | 3.59 | 0.45 | 130 |
| 55 | RUS | D | 1 | 2.27 | 6840 | 3.61 | 0.79 | 51 |
| 56 | RUS | D | 1 | 3.54 | <10 | 3.69 | 0.26 | 84 |
| 57 | RUS | D | 1 | 7.11 | <10 | 3.65 | 0.58 | 88 |
| 58 | RUS | D | 1 | 5.22 | 11 | 3.65 | 0.24 | 103 |
| 59 | RUS | D | 1 | 6.08 | 454 | 3.73 | 0.25 | 54 |
| 60 | RUS | D | 1 | 2.68 | 81 | 3.48 | 0.55 | 64 |
| 61 | RUS | D | 1 | 5.02 | <10 | 4.23 | 0.09 | 101 |
| 62 | RUS | D | 1 | 5.16 | <10 | 4.00 | 0.53 | 57 |
| 63 | RUS | D | 1 | 5.75 | <10 | 3.92 | 0.29 | 80 |
| 64 | RUS | D | 1 | 6.12 | <10 | 3.69 | 0.34 | 52 |
| 65 | RUS | D | 1 | 6.36 | 274 | 3.65 | 0.49 | 184 |
| 66 | RUS | D | 1 | 6.61 | 22 | 4.30 | 0.29 | ND |
| 67 | RUS | D | 1 | 5.04 | 14 | 3.89 | 0.28 | 25 |
| 68 | RUS | D | 1 | 4.46 | 12 | 3.59 | 0.31 | 40 |
| 69 | RUS | A | 1 | 6.14 | <10 | 3.26 | 0.38 | 239 |
| 70 | RUS | D | 1 | 5.81 | 737 | 3.93 | 0.51 | ND |
| 71 | RUS | D | 1 | 5.62 | 16 | 3.99 | 0.30 | ND |
| 72 | RUS | D | 1 | 4.89 | <10 | 2.74 | 0.24 | 136 |
| 73 | RUS | D | 1 | 4.81 | 2152113 | 4.36 | 0.41 | ND |
| 74 | RUS | D | 1 | 5.52 | <10 | 3.54 | 0.25 | 76 |
| 75 | RUS | D | 1 | 4.08 | 56 | 3.82 | 0.40 | 43 |
| 76 | RUS | D | 1 | 4.34 | 16 | 3.67 | 0.25 | ND |
| 77 | RUS | D | 1 | 5.11 | 54 | 4.00 | 0.22 | ND |
| 78 | RUS | D | 1 | 5.23 | <10 | 4.43 | 0.18 | 77 |
| 79 | RUS | D | 1 | 4.82 | 17 | 3.64 | 0.28 | ND |
| 80 | DEU | A | 1 | 5.65 | 20 | 3.34 | 0.50 | 120 |
| 81 | DEU | D | 1 | 4.52 | <10 | 3.23 | 0.32 | 55 |
| 82 | DEU | D | 1 | 6.02 | 23 | 4.26 | 0.26 | 74 |
| 83 | DEU | D | 1 | 5.52 | <10 | 3.87 | 0.16 | 77 |
| 84 | DEU | D | 1 | 4.44 | 518 | 4.28 | 0.21 | 114 |
| 85 | DEU | D | 1 | 5.44 | <10 | 2.92 | 0.20 | 254 |
| 86 | DEU | D | 1 | 5.39 | 23 | 4.08 | 0.19 | 74 |
| 87 | DEU | D | 1 | 4.49 | <10 | 3.95 | 0.16 | 74 |

| | | | | | | | | |
|---|---|---|---|---|---|---|---|---|
| 88 | DEU | D | 1 | 5.74 | <10 | 4.15 | 0.20 | 197 |
| 89 | DEU | D | 1 | 6.83 | <10 | 4.04 | 0.74 | 63 |
| 90 | DEU | D | 1 | 3.60 | 11 | 3.18 | 0.24 | 124 |
| 91 | DEU | D | 1 | 5.01 | 21 | 3.95 | 0.23 | 113 |
| 92 | DEU | D | 1 | 3.49 | <10 | ND | 0.30 | ND |
| 93 | DEU | D | 1 | 6.32 | 166 | ND | 0.35 | ND |
| 94 | DEU | D | 1 | 4.30 | <10 | 2.89 | 0.65 | 289 |
| 95 | DEU | D | 1 | 5.78 | 42 | 4.20 | 0.52 | 247 |
| 96 | DEU | E | 5 | 4.54 | 27 | 3.83 | 0.36 | ND |
| 97 | DEU | D | 1 | 4.54 | <10 | 2.32 | 0.29 | 128 |
| 98 | DEU | D | 1 | 5.33 | <10 | 2.76 | 0.25 | 266 |
| 99 | ITA | D | 1 | 3.61 | 170 | 2.34 | 0.35 | 63 |
| 100 | ITA | D | 1 | 5.22 | 23 | 3.56 | 0.22 | 57 |
| 101 | ITA | D | 1 | 5.22 | 18 | 3.30 | 0.39 | 90 |
| 102 | ITA | D | 1 | 7.00 | 13 | 3.36 | 0.80 | 102 |
| 103 | ITA | D | 1 | 5.84 | <10 | 3.80 | 0.23 | 65 |
| 104 | ITA | D | 1 | 5.38 | 10 | 4.00 | 0.43 | 59 |
| 105 | ITA | D | 1 | 6.41 | 14 | 4.11 | 0.33 | 131 |
| 106 | ITA | D | 1 | 5.41 | <10 | 3.18 | 0.39 | 191 |
| 107 | ITA | D | 1 | 5.46 | 12 | 4.04 | 0.52 | 36 |
| 108 | ITA | D | 1 | 7.21 | 35 | 4.20 | 1.08 | 245 |
| 109 | ITA | D | 1 | 6.21 | <10 | 3.91 | 0.47 | 146 |
| 110 | ITA | D | 1 | 3.73 | <10 | 3.51 | 0.33 | 116 |
| 111 | ITA | D | 1 | 3.26 | <10 | 3.79 | 0.22 | 76 |
| 112 | ITA | D | 1 | 5.93 | <10 | 3.69 | 0.36 | 161 |
| 113 | ITA | D | 1 | 4.28 | <10 | 3.83 | 0.30 | 39 |
| 114 | ITA | D | 1 | 4.75 | 61 | 3.65 | 0.41 | ND |
| 115 | SWE | A | 1 | 4.48 | 42 | 3.49 | 0.50 | ND |
| 116 | CIV | A | 1 | ND | ND | ND | 0.29 | 144 |
| 117 | CIV | A | 5 | ND | <10 | ND | 0.23 | 36 |
| 118 | CIV | E | 5 | ND | 490 | ND | 0.55 | 42 |
| 119 | MDA | A | 1 | ND | <10 | ND | 0.18 | 206 |
| 120 | RUS | D | 1 | ND | <10 | ND | 0.10 | 19 |
| 121 | CMR | E | 1 | ND | 15100 | ND | 0.79 | 72 |
| 122 | ROU | A | 1 | ND | ND | ND | 0.41 | 226 |
| 123 | RUS | D | 2 | ND | ND | ND | 0.24 | ND |
| 124 | RUS | A | 1 | ND | ND | ND | 0.27 | ND |
| 125 | FRA | C | 1 | ND | <20 | ND | 0.13 | 60 |
| 126 | FRA | E | 1 | ND | 27 | ND | 0.66 | 35 |
| 127 | ITA | B | 1 | ND | ND | ND | 0.45 | ND |
| 128 | ITA | A | 1 | ND | ND | ND | 0.82 | ND |

[a] RUS = Russia; FRA = France; DEU = Germany; ITA = Italy; SWE = Sweden; CIV = Cote D'Ivoire; MDA = Moldova; CMR = Cameroon; ROU = Romania
