# Supplementary material for: Broad-spectrum activity of bulevirtide against clinical isolates of HDV and recombinant pan-genotypic combinations of HBV/HDV
Source: JHEP Rep. 2023 Aug 22;5(11):100893. doi: 10.1016/j.jhepr.2023.100893 (PMC10622701; doi:10.1016/j.jhepr.2023.100893)
Supplement: Multimedia component 4 [file mmc4.pdf]

Broad-spectrum activity of bulevirtide against clinical isolates of HDV and recombinant pan-genotypic combinations of HBV/HDV

Roberto Mateo,[1,*] Simin Xu,[1] Alex Shornikov,[1] Tahmineh Yazdi,[1] Yang Liu,[1] Lindsey May,[1] Bin Han,[1] Dong Han,[1] Ross Martin,[1] Savrina Manhas,[1] Christopher Richards,[1] Caleb Marceau,[1] Thomas Aeschbacher,[1] Silvia Chang,[1] Dmitry Manuilov,[1] Julius Hollnberger,[2,3] Stephan Urban,[2,3] Tarik Asselah,[4] Dzhamal Abdurakhmanov,[5] Pietro Lampertico,[6,7] Evguenia Maiorova,[1] Hongmei Mo[1]

[1]Gilead Sciences Inc., Foster City, CA, USA; [2]Department of Infectious Diseases, Molecular Virology, University Hospital Heidelberg, Heidelberg, Germany; [3]German Center for Infection Research (DZIF), Heidelberg Partner Site, Heidelberg, Germany; [4]Université de Paris-Cité, Centre de Recherche sur l'Inflammation, INSERM UMR 1149, Hôpital Beaujon, Department of Hepatology, AP-HP, Clichy, France; [5]Sechenov First Moscow State Medical University, Moscow, Russia; [6]Division of Gastroenterology and Hepatology, Foundation IRCCS Ca' Granda Ospedale Maggiore Policlinico, Milan, Italy; [7]CRC 'A. M. and A. Migliavacca' Center for Liver Disease, Department of Pathophysiology and Transplantation, University of Milan, Milan, Italy

JHEP Reports **2023.** https://doi.org/10.1016/j.jhepr.2023.100893

Background & Aims: Bulevirtide (BLV) is a small lipopeptide agent that specifically binds to the sodium taurocholate cotransporting polypeptide (NTCP) bile salt transporter and HBV/HDV receptor on the surface of human hepatocytes and inhibits HDV and HBV entry. As a satellite virus of HBV, HDV virions are formed after assembly of HDV RNA with the HBV envelope proteins (HBsAg). Because both viruses exist as eight different genotypes, this creates a potential for high diversity in the HBV/HDV combinations. To investigate the sensitivity of various combinations of HBV/HDV genotypes to BLV, clinical and laboratory strains were assessed.

Methods: For the laboratory strains, the different envelopes from HBV genotypes A through H were combined with HDV genotypes 1–8 in cotransfection assays. Clinical plasma isolates were obtained from clinical studies and academic collaborations to maximise the diversity of HBV/HDV genotypes tested.

Results: The mean BLV EC_{50} against HDV laboratory strains ranged from 0.44 to 0.64 nM. Regardless of HBV and HDV genotypes, the clinical isolates showed similar sensitivities to BLV with mean values that ranged from 0.2 to 0.73 nM.

Conclusions: These data support the use of BLV in patients infected with any HBV/HDV genotypes.

Impact and implications: This study describes the potent activity of BLV against multiple laboratory strains spanning all HBV/HDV A–H/1–8 genotype combinations and the most diverse collection of HDV clinical samples tested to date, including HBV/HDV genotype combinations less frequently observed in the clinic. Overall, all isolates and laboratory strains displayed similar *in vitro* nanomolar sensitivity to BLV. This broad-spectrum antiviral activity of BLV has direct implications on potential simplified treatment for any patient infected with HDV, regardless of genotype, and supports the new 2023 EASL Clinical Practice Guidelines on HDV that recommend antiviral treatment for all patients with CHD.

© 2023 The Author(s). Published by Elsevier B.V. on behalf of European Association for the Study of the Liver (EASL). This is an open access article under the CC BY license (http://creativecommons.org/licenses/by/4.0/).

Introduction

Chronic hepatitis D (CHD) is the most severe form of viral hepatitis, with rapid progression of liver-related diseases and high rates of development of hepatocellular carcinoma.[1,2] At least 12 million people worldwide have experienced HDV infection, although the exact prevalence remains controversial because of unreliable assessment of the infection and the lack of real-world surveillance.[3] Recent data have shown that HDV continues to spread in low-income countries with a prevalence that has remained stable or increased in certain regions.[4] Therefore, the real number of patients infected with HDV may have been severely underestimated.[5] With limited therapeutic interventions available, CHD disease remains an urgent but unmet medical need.[6,7]

HDV, the causative agent of CHD, is a defective small RNA virus that requires the HBsAg for its complete infection and transmission.[8–11] Eight genotypes of HDV exist, with genotype 1 (HDV-1) being the most common globally and in North America.[8] Whereas HDV-1 is the most prevalent and distributed worldwide, HDV-2 is restricted to Southeast Asia.[12] HDV-3, the most distantly related genotype, is common in northern areas of South America, whereas HDV-4 is found in Japan and China.[12] HDV-5, -6, -7, and -8 are prevalent in African countries.[12]

HDV RNA can replicate in the liver in the absence of HBV.[11] This tissue specificity is based on the hepatocyte-specific expression of the sodium taurocholate cotransporting polypeptide (NTCP) receptor, a bile acid transporter that also acts as an HBV/HDV receptor.[13] For secretion and *de novo* entry, newly

Keywords: hepatitis delta virus; hepatitis; Bulevirtide.
Received 3 May 2023; received in revised form 20 July 2023; accepted 7 August 2023; available online 22 August 2023
* Corresponding author. Address: Gilead Sciences Inc., 333 Lakeside Drive, Foster City, 94404 CA, USA. Tel.: +1 650 200 5345.
E-mail address: roberto.mateo@gilead.com (R. Mateo).

ELSEVIER EASL The Home of Hepatology

synthesised HDV RNA/protein complexes require all three forms of HBsAg, namely, large (L), middle (M), and small (S), which collectively compose the HBV envelope.[11] Notably, HBV genotypes have different geographical distributions: genotype A (GTA), Sub-Saharan Africa and Asia; GTB and GTC, Asia; GTD, Asia, Africa, and Europe; GTE, Sub-Saharan Africa; and GTF–H, Latin America.[14] In HDV-infected patients, however, the distribution of HBV genotypes has not been thoroughly investigated.

Synthetic PreS1 lipopeptides from diverse HBV genotypes differ in their ability to interfere with the binding of HBV to NTCP.[15] This argues that HDV particles containing envelope proteins from different HBV genotypes might vary in their infection efficacy, spreading abilities, and pathogenesis in patients. Therefore, the study of the multiple HBV/HDV combinations and their susceptibilities to treatment remains of great importance to assess coverage of circulating HDV viruses.[16]

Bulevirtide (BLV), formerly known as Myrcludex B, is a 47-amino acid, N-terminally myristoylated, HBV large envelope (L) protein–derived lipopeptide that binds specifically to NTCP and acts as a potent, highly selective entry inhibitor of HDV and HBV into hepatocytes.[17,18] In April 2023, BLV 2 mg, administered as a daily s.c. injection, was granted full marketing authorization by the EMA for the treatment of CHD and compensated liver disease. Several clinical studies have shown its safety and efficacy at reducing HDV viral load and normalising alanine aminotransferase levels.[19–24] Unfortunately, limited data on the efficacy of BLV on different HBV/HDV genotype combinations exist, as most participants enrolled in these studies were HBV/HDV D/1, with some A/1 and a few E/5. In the clinic, it is unlikely that all possible combinations of HBV/HDV genotypes exist, making coverage evaluation of BLV for all potential genotype combinations unattainable by analysing patient samples only.

In this study, the *in vitro* antiviral activity of BLV against laboratory-derived HDV-1 to HDV-8 viruses enveloped with HBV GTA–H (81 recombinant viruses) was assessed. Similarly, a panel of 128 clinical plasma isolates of diverse combinations of HDV/HBV genotypes were tested for their inhibitory properties to BLV.

Materials and methods

Sensitivity assays of BLV were performed at Gilead Sciences, Inc. (Foster City, CA, USA).

Clinical isolates

A panel of patient plasma samples from 115 HDV-infected participants enrolled in Gilead-sponsored clinical studies and treated with BLV (studies MYR204 [ClinicalTrials.gov, NCT03852433; EudraCT 2019-001485-15][25] and MYR301 [ClinicalTrials.gov, NCT03852719; EudraCT 2019-001213-17])[26] were tested for *in vitro* sensitivity against BLV. In addition, 13 plasma samples from participants infected with HDV from less common HBV genotypes (GTA, GTB, GTC, and GTE) were studied for their sensitivity to BLV. These samples come from participants from six different countries of birth and were obtained through external collaborators.

All studies were conducted in accordance with the Declaration of Helsinki, Good Clinical Practice guidelines, and local regulatory requirements. All patients provided written informed consent.

Compounds

BLV was synthesised by Gilead.

Cell culture

HuH7 cells were obtained from ReBLikon GmbH. Cells were maintained in DMEM/F12 supplemented with 10% heat-inactivated FBS (Hyclone) and 1× penicillin–streptomycin–glutamine (Life Technologies).

Primary human hepatocytes (PHHs) were obtained from BioIVT. Cells were plated with William's Medium E (Thermo Scientific) supplemented with Hepatocyte Plating Supplement Pack and maintained in William's Medium E with 2% FBS, 1.5% DMSO (Sigma-Aldrich), and Hepatocyte Maintenance Supplement Pack (Gibco). All assays in this report were performed using a single PHH donor (KBK).

Construction of HBV envelope expression constructs

HBV envelope for GTA–H

The eight constructs carrying HBV GTA–H envelopes are gifts from Dr. Stephan Urban. In brief, the HB2.7 subgenomic fragments were amplified by PCR from eight constructs containing HBV GTA–H sequences. The accession numbers for these HBV GTA–H sequences are MN645903–MN645910. The HB2.7 subgenomic fragments were then inserted into lentiviral production plasmid pLX304. Detailed information about these constructs is described by Wang *et al.*[16]

HBV envelope polymorphisms

A total of 7,427 sequences from public databases and internal sources comprising HBV GTA–H (6,768 sequences were GTA–D) were analysed to identify polymorphisms within the BLV region of HBV envelope for each genotype. For GTA–D, HBV envelope polymorphism constructs were designed to cover the most common polymorphisms of the BLV region. A total of 24 HBV envelope polymorphism constructs were generated: two for GTA, 10 for GTB, 10 for GTC, and two for GTD. Table S2 shows the sequence alignment of representative sequences from HBV GTA–H with the BLV sequence. The HBV envelope constructs containing polymorphic changes were generated by synthesising a 2.7-kb HBV fragment containing HBV envelope from clinical isolates across GTA–D and cloning into pLX304 plasmids.

HDV constructs

The 1.1-fold antigenomic cDNA sequences of HDV-1 (accession number U81989), HDV-2 (accession number X60193), HDV-3 (accession number L22063), HDV-4 (accession number AB118847), HDV-5 (accession number AM183331), HDV-6 (accession number AM183329), HDV-7 (accession number AM183333), and HDV-8 (accession number AM183327) were gifts from Dr. Stephan Urban. More detailed information about these constructs is described by Wang *et al.*[16]

Transient transfection

HDVs were produced by cotransfection of HuH7 cells with the corresponding HDV plasmids (pcDNA3.1-HDV-1 to pcDNA3.1-HDV-8) and HBV plasmids pLX304-HB2.7-A to pLX304-HB2.7-H using Lipofectamine 3000 as previously described.[16] In brief, HuH7 cells were seeded in T75 flasks at 4×10^6 cells 1 day before transfection. Sixteen hours after transfection, cells were washed with PBS and fresh medium. Media were replaced every second or third day and collected at Day 8 or 13 for PHH infections. Quantitation of HDV RNA in the supernatant of cells after

transfection of an in-house HDV-1 laboratory strain carrying the HBV GTB envelope (BB-1) was used as a control for transfection efficiency in all experiments.

HDV RNA quantification

HDV RNA was extracted from the supernatant medium using the QIAamp viral RNA Mini KIt (QIAGEN, Cat# 52904) according to the manufacturer's instructions. In brief, the viral supernatant was first lysed under highly denaturing conditions and loaded onto the QIAamp Mini spin column. The RNA bound to the membrane and washed with two different wash buffers to remove protein, nucleases, and other contaminants and inhibitors. RNA was eluted in nuclease-free water and proceeded to the next step or stored at -80 °C freezer for later use.

RNA then was treated with DNase I (Thermo Fisher, Cat# 18068015) to remove any residual DNA. Quantitative real-time PCR for HDV RNA was performed using TaqMan^{TM} Fast Virus 1-Step Master Mix (Thermo Fisher, Cat# 4444434) on a QuantStudio Fast Real-Time PCR system (Thermo Fisher). Amplification primers are as follows: HDV forward, GCGCCGGCYGGGCAAC; HDV reverse, TTCCTCTTCGGGTCGGCATG; and HDV probe, CGCGGTCCGACCTGGGCATCCG. RNA copies per millilitre were calculated against a standard curve.

EC_{50} determination

EC_{50} values were determined by seeding PHHs in 96-well plates at a density of 60,000 cells/well. One day after seeding, PHHs were pretreated with 10 replicate dilutions of BLV for 0.5 to 1 h before virus inoculation. After BLV incubation, PHHs were infected with 2 µl of clinical plasma or 40 µl of an HDV laboratory strain, unless otherwise indicated, in the presence of 4% PEG8000 (Sigma-Aldrich) and 1.5% v/v DMSO and kept in culture for 5 days. An in-house HDV-1 laboratory strain carrying the HBV GTB envelope (BB-1) was included as a control. HDV antigen (HDAg)-positive cells were stained and quantified as described in the HDAg Staining section.

HDAg staining

Immunofluorescence staining against HDAg was performed at Day 5 post infection. In brief, PHH cells cultured in white, collagen-coated, clear 96-well plates were fixed in 4% paraformaldehyde for 30 min, followed by 30 min of incubation in blocking buffer (PBS, 0.3% Triton, 3% BSA, 10% FBS). After washing with PBS, cells were incubated for 1 h with an anti-HDAg mouse monoclonal antibody (Gilead) diluted in incubation buffer (PBS, 0.3% Triton, 3% BSA). Then, cells were washed and incubated with Hoescht and secondary goat antimouse (Invitrogen) antibody for 1 h. After extensive washing, the percentage of HDAg-positive cells compared with total cell numbers was calculated using software HSC Studio Cellomics Scan (version 6.6.2, Thermo Scientific).

Statistical analysis

Data were calculated using nonlinear regression analysis GraphPad Prism 8 software package (IDBS, Emeryville, CA, USA). The concentration of BLV for which there was 50% decrease in HDV antigen production was expressed as a mean EC_{50} value from at least two independent experiments.

Results

Establishment and optimisation of an assay for robust production of infectious HDV viral particles of diverse genotypes

Genotype-specific studies of the infectivity of HDV-1 to HDV-8 enveloped with L-HBsAg from HBV GTA–H have been described before using HuH7-NTCP cells.[16] In this assay, HuH7-NTCP cells were plated into large flasks and cotransfected with a mixture of HDV and HBV plasmids. Cultures were incubated and supernatants were harvested and tittered on Days 8 and 13 to study the kinetics of production of infectious HDV (Fig. 1). As shown in Fig. 1B using HDV-3, viral titres were generally similar or increased slightly from Day 8 to Day 13. Regardless, by Day 8, all combinations of HDV genotypes with HBV GTA–H had produced enough infectious virus ($\sim 10^4$ copies/ml) to determine their sensitivity to BLV. Based on this information, all transfections were carried out for 8 days unless otherwise indicated.

Establishment of an HDV viral infectivity assay using PHHs

Viruses generated after cotransfection and collected on Day 8 were used to infect PHHs. Five days later, PHH monolayers were fixed, and HDAg was quantified by immunofluorescence (Fig. 2A). Combinations of HBV GTA–H with HDV-1 to HDV-8 resulted in differences in rates of HDAg positivity higher than ~100-fold depending on the particular combination (Fig. 2B–D and Table S1). However, most combinations displayed sufficient infectivity to assess their sensitivity to BLV, even with infectivity values as low as 0.03% (HBV/HDV H/7). Specifically, HBV/HDV genotype combinations E/7, E/5, and B/1 had the highest percentage of HDAg positivity in PHHs, with values of 25%, 20%, and 19%, respectively. On the contrary, HBV/HDV genotype combinations F/2 and H/7 had the lowest measurable values of 0.02% and 0.03%, respectively. Seven rare HBV/HDV genotype combinations resulted in no measurable fluorescence signal in the PHH assay: A/4, C/4, D/4, F/4, H/4, F/6, and H/6. Overall, a measurable percentage of cells positive for HDAg staining could be determined for 89% (57/64) of viruses generated.

Sensitivity to BLV of HDV laboratory strains comprising HDV-1 to HDV-8 enveloped with HBV GTA–H

To determine the BLV sensitivity of HDV-1 to HDV-8 enveloped with HBV GTA–H, viruses were generated *in vitro* and treated with BLV in the PHH infection assay as described above. Despite differences in the *infectivity* (percentage of HDAg-positive cells) for the various combinations, BLV potently inhibited HDV infection in all the 57 HDV/HBV combinations that produced enough infectious virus in this assay. Similar EC_{50} values across genotypes were observed (Table 1). However, seven laboratory strains resulted in assay failure as no measurable signal for HDAg staining was detected: HDV-4 with HBV GTA, GTC, GTD, GTF, and GTH envelopes and HDV-6 with HBV GTF and GTH envelopes. Overall, BLV EC_{50} values could be determined for 89% (57/64) of the viruses generated (Table 1).

The mean EC_{50} of BLV against HDV-1 to HDV-8 enveloped with HBsAg from HBV GTA-D ranged from 0.38 to 0.71 nM. Similarly, for HDV-1 to HDV-8 enveloped with HBsAg from HBV GTE–H, the mean EC_{50} of BLV ranged from 0.34 to 0.68 nM.

Across HDV-1 to HDV-8 enveloped with HBV GTA–H envelopes, the mean EC_{50} of BLV ranged from 0.44 to 0.64 nM. Taken together, BLV showed similarly potent activity against all eight

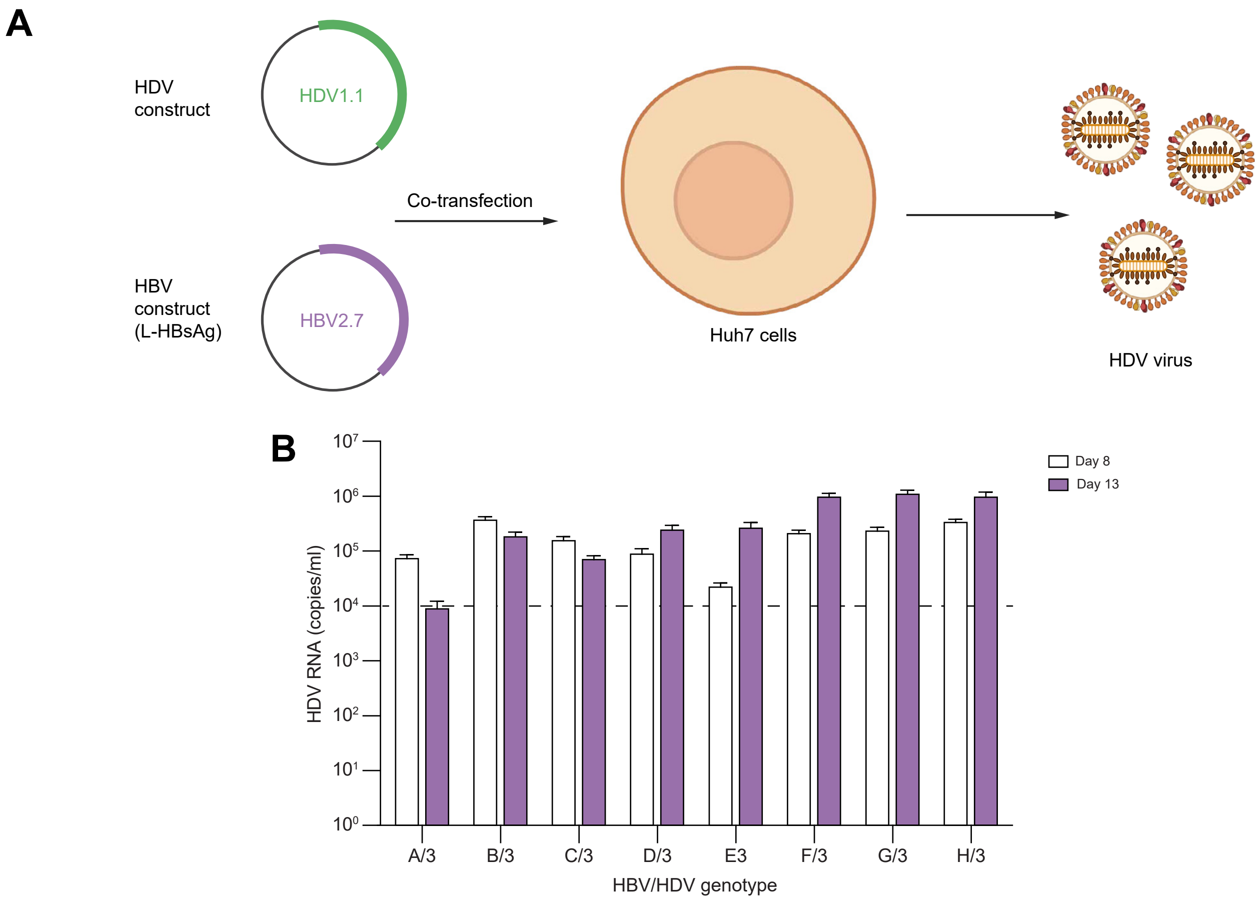

Fig. 1. Production of infectious HDV. (A) Cells were seeded and used to produce HDVs by cotransfection with HDV and HBV plasmids. Sixteen hours after transfection, cells were washed, and supernatant medium was refreshed and collected at Days 8 or 13 and cleared with centrifugation before HDV RNA quantitation. (B) Supernatants were collected, and RNA was extracted and quantified using qRT-PCR. Production of HDV genotype 3 cotransfected with PreS1 from HBV GTA–H is shown as a representative example. An average of three replicate experiments is shown. Error bars indicate SD. BLV, bulevirtide; GTA–H, genotypes A–H; HDAg, HDV antigen; qRT-PCR, quantitative real-time PCR.

HDV genotypes and eight HBV genotypes with a very narrow range of EC_{50} values.

Activity of BLV against HDV-1 with representative PreS1 sequences from HBV GTA–H and the most common polymorphisms in HBV GTA–D

To determine the impact of PreS1 polymorphisms on sensitivity to BLV, polymorphic analyses of 7,427 HBV sequences from GTA–H obtained from public databases and Gilead internal sources were conducted. A high degree of conservation of the 47-amino acid-long peptide sequence that corresponds to the BLV sequence was found, with a remarkable conservation of the sequence from positions 9-NPGLFFP-15, which is crucial for binding. Table S2 shows the alignment of representative sequences from HBV GTA–H with the BLV sequence. Among the most common genotypes GTA–D, GTC was the closest to the BLV sequence with only one amino acid change (K57Q). GTA and GTB had four and six amino acid changes, respectively, and GTD had eight compared with the BLV sequence. Importantly, the sensitivity to BLV of HDVs containing these sequences were similar for the common GTA–D with EC_{50} values ranging from 0.46 to 0.90 nM (Table S2). The remaining HBV GTE–H had between eight and nine amino acid changes compared with the BLV sequence; however, their EC_{50} values were within assay variation and remained similar to those of the other genotypes with values ranging from 0.28 to 0.85 nM. Overall, no significant differences across different genotypes were observed for any of the viruses tested.

Next, a sequence analysis was conducted to determine the most common amino acid variations in the PreS1 sequence found in clinical isolates from HBV GTA–D (Table S3). To further evaluate the activity of BLV against these multiple variants identified, *in vitro* HDV-1 laboratory strains were generated. Given that HDV clinical isolates with HBV GTB and GTC envelopes were underrepresented in our BLV clinical isolates, a bigger subset of HBV GTB and GTC envelope constructs compared with GTA and GTD were generated with 10 HBV GTB and 10 HBV GTC envelope constructs each, as well as two HBV GTA and GTD envelope constructs each. Regardless of polymorphisms, the mean EC_{50} of BLV for HDV-1 enveloped with multiple strains of HBV GTA, GTB, GTC, and GTD were 0.57, 0.61, 0.42, and 0.32 nM, respectively, very similar to those obtained for HDVs enveloped with PreS1 containing representative sequences of HBV GTA, GTB, GTC, and GTD (0.65, 0.90, 0.46, and 0.49 nM, respectively)

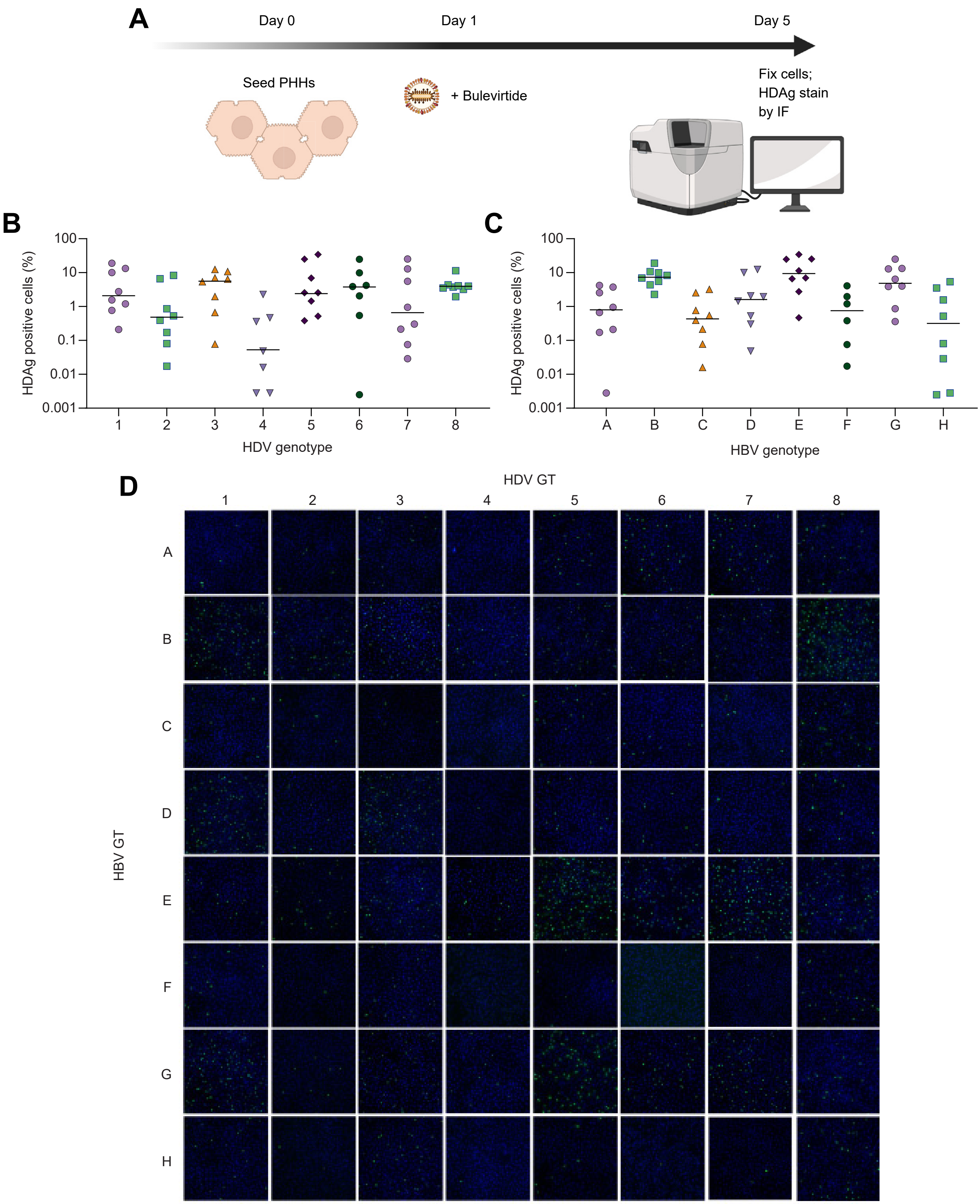

Fig. 2. ***In vitro*** **HDV phenotypic assay in PHHs.** (A) PHHs were seeded and pretreated with several dilutions of BLV before virus inoculation. Then, they were infected with clinical plasma or an HDV laboratory strain and kept in culture for 5 days. At that time, IF staining against HDAgs was performed, and the percentage of positive cells was calculated using software HCS Studio Cellomics Scan. (B) Percentage of HDAg-positive cells by infecting HDV genotypes 1–8. Each data point represents the average of two independent experiments. Bars show median values. (C) Percentage of HDAg-positive cells stratified by infecting HBV genotypes A–

Table 1. Antiviral activity of BLV against HDV-1 to HDV-8 enveloped with HBV GTA–H.

	BLV EC_{50} (nM)									
HBV GT	HDV-1	HDV-2	HDV-3	HDV-4	HDV-5	HDV-6	HDV-7	HDV-8	HBV mean	HBV median
GTA	0.65 ± 0.03	0.44 ± 0.06	0.32 ± 0.05	n.a.	0.52 ± 0.03	0.48 ± 0.08	0.59 ± 0.11	0.43 ± 0.09	0.49	0.48
GTB	0.90 ± 0.29	0.53 ± 0.01	0.50 ± 0.06	0.71 ± 0.24	0.84 ± 0.04	0.66 ± 0.03	0.82 ± 0.03	0.73 ± 0.06	0.71	0.72
GTC	0.46 ± 0.17	0.59 ± 0.13	0.32 ± 0.06	n.a.	0.32 ± 0.02	0.49 ± 0.02	0.46 ± 0.10	0.93 ± 0.24	0.51	0.46
GTD	0.49 ± 0.04	0.23 ± 0.06	0.36 ± 0.06	n.a.	0.52 ± 0.05	0.33 ± 0.13	0.38 ± 0.12	0.38 ± 0.05	0.38	0.38
GTE	0.85 ± 0.09	0.39 ± 0.04	0.54 ± 0.05	0.65 ± 0.01	0.76 ± 0.13[†]	0.65 ± 0.03[†]	0.80 ± 0.05	0.64 ± 0.11	0.66	0.65
GTF	0.28 ± 0.09	0.38[*]	0.30 ± 0.11	n.a.	0.34 ± 0.03	n.a.	0.24 ± 0.02	0.52 ± 0.10	0.34	0.32
GTG	0.57 ± 0.09	0.68 ± 0.07	0.76 ± 0.01	0.56 ± 0.01	0.73 ± 0.08[†]	0.67 ± 0.18	0.86 ± 0.02	0.59 ± 0.01	0.68	0.68
GTH	0.45 ± 0.03	0.61 ± 0.18	0.39 ± 0.04	n.a.	0.34 ± 0.07	n.a.	0.31 ± 0.06	0.56 ± 0.12	0.44	0.42
HDV mean	0.58	0.48	0.44	0.64	0.55	0.55	0.56	0.60		
HDV median	0.53	0.49	0.38	0.65	0.52	0.57	0.53	0.58		

HBV/HDV GT combinations were produced by transfection of HuH7 cells and phenotyped against BLV in replicate experiments in a PHH infectivity assay. Mean and median values are shown.
BLV, bulevirtide; GT, genotype; GTA–H, genotypes A–H; HDV-1 to HDV-8, HDV genotypes 1–8; n.a., not applicable owing to low infectivity; PHH, primary human hepatocyte.
[*] n = 1 owing to very low infectivity.
[†] EC_{50} was averaged in two independent experiments with 40 and 4 μl of input viral inoculum.

(Table 2 and Fig. 3A). Thus, BLV maintained similar and potent inhibition of HDV-1 enveloped with HBV GTA–D laboratory strains containing the major polymorphisms in PreS1.

Analysis of the relationship between infectivity and sensitivity to BLV of HDV laboratory strains

Next, the relationship between the viral infectivity (percentage of HDAg-positive PHHs) of all HDV laboratory strains generated containing all the combinations of HBV/HDV and their *in vitro* sensitivity to BLV was investigated. As shown in Fig. 3, regardless of the genotype or their infectivity, all laboratory strains showed similar sensitivity to BLV (Fig. 3C). Similarly, when HDVs containing the most common polymorphisms observed in the PreS1 region of HBV GTA–D were investigated for their sensitivity to BLV, tight EC_{50} values similar to those of the corresponding HBV representative sequences for each genotype (denoted as stars) were observed (Fig. 3B).

In summary, sensitivity to BLV was not affected by either the HBV PreS1 sequence in the envelope of the HDVs tested or their infectivity in PHHs, with EC_{50} values that ranged between 0.17 and 0.93 nM (Table 2).

Establishment of an HDV phenotyping assay for clinical samples from patients with CHD

Plasma samples from participants with CHD were analysed for their *in vitro* infectivity and sensitivity to BLV in an assay similar to the one developed for testing HDV laboratory strains. In brief, 2 μl of patient plasma was used to infect a monolayer of freshly plated PHH cells. Five days later, cells were fixed and stained for HDAg, and the percentage of infected cells was calculated using fluorescent cell imaging. The assay showed robust infection of samples with high reproducibility of infection and EC_{50} values (data not shown). Importantly, there was a significant statistical correlation (Pearson correlation; p <0.0001, r^2 = 0.733) between levels of PHH infection (percentage of HDAg-positive cells) and levels of HDV RNA, suggesting that this faster image-based assay could be used as a surrogate for HDV replication and infectivity and to determine sensitivity to BLV (Fig. 4A).

Activity of BLV against clinical isolates of various HBV/HDV genotypes from participants with CHD

The activity of BLV against 128 clinical isolates was evaluated in the PHH infectious system described above. The majority of clinical isolates tested (n = 107) were HBV/HDV D/1 and within a narrow range of the mean EC_{50} of 0.39 nM (Fig. 4B and Table 3). Ten participants were HBV/HDV A/1 with a mean EC_{50} of 0.47 nM. Four participants were infected with HBV/HDV E/5, and the clinical isolates from these participants were sensitive to BLV with a mean EC_{50} of 0.50 nM (Fig. 4B and Table 3). The patient isolates from the HBV/HDV combinations B/1, C/1, E/1, A/6, D/2, and A/5 were also tested, with mean EC_{50} values that ranged from 0.20 to 0.73 nM (Fig. 4B and Table 3).

Most clinical isolates tested were carrying HBV GTD envelopes (n = 108). Notably, the EC_{50} value of these isolates were within a narrow range with the 95th percentile of EC_{50} values being only approximately four-fold higher than the 5th percentile of EC_{50} values.

Taken together, these data demonstrate that BLV maintains effective inhibition of HDV infection across genotypes of HDV and HBV.

Analysis of the relationship between viral infectivity and sensitivity to BLV in clinical samples from participants with CHD

An analysis was performed with all available HDV clinical specimens, comparing their viral infectivity and *in vitro* sensitivity to BLV. Despite differences in infectivity, all clinical isolates remained susceptible to BLV with mean EC_{50} values that ranged from 0.20 to 0.73 nM (Fig. 4C and Table 3). Overall, there was no major effect of viral input on EC_{50} value determination, which allowed for testing of the majority of clinical samples regardless of viral load (128/141, 91%).

H tested. Each data point represents the average of two independent experiments. Bars show median values. (D) Representative fluorescence images of HDAg staining on Day 5 post infection for all HBV/HDV genotype combinations. Each assay was performed at least in two independent experiments. BLV, bulevirtide; GT, genotype; HDAg, HDV antigen; IF, immunofluorescence; PHH, primary human hepatocyte.

Table 2. Antiviral activity of BLV against multiple strains of HBV GTA–D enveloped with HDV-1.

	HBV GTA		HBV GTB		HBV GTC		HBV GTD	
	HBV construct	EC_{50} (nM)	HBV construct	EC_{50} (nM)	HBV construct	EC_{50} (nM)	HBV construct	EC_{50} (nM)
EC_{50} (nM) of individual viral strains	GTA*	0.65 ± 0.03	GTB*	0.90 ± 0.29	GTC*	0.46 ± 0.17	GTD*	0.49 ± 0.04
	GTA-1	0.17 ± 0.05	GTB-1	0.47 ± 0.06	GTC-1	0.47 ± 0.07	GTD-1	0.29 ± 0.03
	GTA-2	0.88 ± 0.10	GTB-2	0.74 ± 0.19	GTC-2	0.21 ± 0.01	GTD-2	0.20 ± 0.01
	–	–	GTB-3	0.55 ± 0.02	GTC-3	0.71 ± 0.30	–	–
	–	–	GTB-4	0.42 ± 0.19	GTC-4	0.24 ± 0.01	–	–
	–	–	GTB-5	0.41 ± 0.05	GTC-5	0.45 ± 0.08	–	–
	–	–	GTB-6	0.93 ± 0.01	GTC-6	0.28 ± 0.05	–	–
	–	–	GTB-7	0.67 ± 0.04	GTC-7	0.88 ± 0.10	–	–
	–	–	GTB-8	0.55 ± 0.38	GTC-8	0.29 ± 0.04	–	–
	–	–	GTB-9	0.68 ± 0.25	GTC-9	n.a.	–	–
	–	–	GTB-10	0.43 ± 0.07	GTC-10	0.26 ± 0.11	–	–
Mean EC_{50} (nM)	GTA	0.57	GTB	0.61	GTC	0.42	GTD	0.33
Median EC_{50} (nM)	GTA	0.65	GTB	0.55	GTC	0.37	GTD	0.29

HBVs/HDVs were produced by transfection of HuH7 cells and were tested for their sensitivity to BLV in PHHs. Mean and median values from replicate experiments are shown. BLV, bulevirtide; GTA–D, genotypes A–D; HDV-1, HDV genotype 1; n.a., not applicable owing to low infectivity; PHH, primary human hepatocyte.
* Representative sequences for each genotype.

In conclusion, similar EC_{50} values for BLV were observed regardless of the HBV/HDV genotype combination of the sample tested.

Analysis of the relationship between HDAg and PreS1 sequence in BLV sensitivity in clinical samples from patients with CHD

Next, a possible correlation between *in vitro* BLV activity and the sequence of the HDAg of all HDV clinical samples available was investigated. Because the majority of the clinical samples grouped to genotypes HBV-D/HDV-1, an HDV-1 consensus sequence from 10 reference sequences was constructed. Compared with this consensus, polymorphisms with several specific variations were identified. Despite the difference in sequence positions, the sensitivity to BLV remained comparable with EC_{50} values that ranged from 0.09 to 1.27 nM (Fig. 5A).

Lastly, the sequence of the homologous BLV region to PreS1 (positions 13–59) of all clinical isolates was directly compared with the BLV sequence. This analysis revealed differences in positions at residues 14, 18, 19, 35, 38, 39, 51, and 54. Therefore, an analysis of the activity of BLV on isolates containing these variants was performed. As shown in Fig. 5, despite sequence differences, there was no significant effect on the sensitivity of these viral variants to inhibition with BLV *in vitro*, with EC_{50} values ranging from 0.13 to 1.27 nM (Fig. 5B).

Overall, despite the high sequence diversity of all the HDV clinical isolates and laboratory strains tested (Fig. 5C), all samples were sensitive to BLV *in vitro* with a very narrow EC_{50} range. This argues for the use of BLV as a therapy with potential broad-spectrum genotypic coverage in patients with CHD.

Discussion

This study describes the broad and potent activity of BLV in 81 recombinant laboratory strains, comprising HDV-1 to HDV-8 in combination with HBV L-HBsAg from HBV GTA–H, as well as a collection of 128 different clinical samples from patients with CHD.

A phenotyping assay using PHHs allowed us to determine the activity of BLV for HDV laboratory strains of genotypes 1, 2, 3, 5, 7, and 8 enveloped with L-HBsAg from HBV GTA–H. Similarly, HDV-4 combinations with HBV GTB (B/4), GTE (E/4), and GTG (G/4) and HDV-6 combinations with HBV GTA–E (A–E/6) and GTG (G/6) were analysed. All of these combinations were highly sensitive to BLV, with mean EC_{50} values that ranged from 0.34 to 0.71 nM. In addition, 24 HDV laboratory strains consisting of the HDV-1 genome enveloped with L-HBsAg containing the most common polymorphisms in HBV GTA–D (two for HBV GTA, 10 for HBV GTB, 10 for HBV GTC, and two for HBV GTD) representing >7,000 sequences from clinical isolates were generated. The corresponding HDVs were all sensitive to BLV with EC_{50} values <1 nM, indicating that the activity of BLV is not affected by the particular HBV PreS1 sequence in the envelope of the HDVs.

A conservation analysis of the PreS1 region performed after sequence alignment of the over 7,000 clinical isolates mentioned above revealed an overall high degree of conservation among all genotypes. In addition, the BLV sequence 9-NPGLFFP-15, crucial for binding to NTCP, was found to be extremely conserved among genotypes except for residue F25L in GTF (16.6% frequency; data not shown). Overall, polymorphisms in PreS1 were located far from the residues critical for NTCP binding and were not consistent across GTA–H. For GTA, only one residue was found to be variable in >5% of the sequences analysed, with GTC being the most different (five residues above the 5% cut-off value). GTB and GTD had two variable residues each. In summary, despite some PreS1 polymorphisms identified within genotypes, all viruses were sensitive to BLV treatment, highlighting the overall broad antiviral activity of BLV.

Similarly, a collection of 128 plasma samples from patients with CHD covering HBV/HDV genotype combinations D/1, D/2, A/1, A/5, A/6, C/1, B/1, E/1, and E/5 were blocked from entry into hepatocytes by BLV, with EC_{50} values that were within a narrow range of 0.1–1.3 nM, with the 95th percentile of EC_{50} values being only two- to five-fold higher than the 5th percentile of EC_{50} values. Sequence analysis of the BLV region of PreS1 in the envelope of these clinical isolates revealed 22 substitutions (22/47, 47%) when compared with the BLV sequence. These sequence variations did not significantly change inhibition by BLV. Although unlikely to affect sensitivity to BLV, 86 substitutions were identified in the HDAg of these clinical isolates and tested against BLV. As expected, they did not have an effect on sensitivity to BLV either (Fig. 5A).

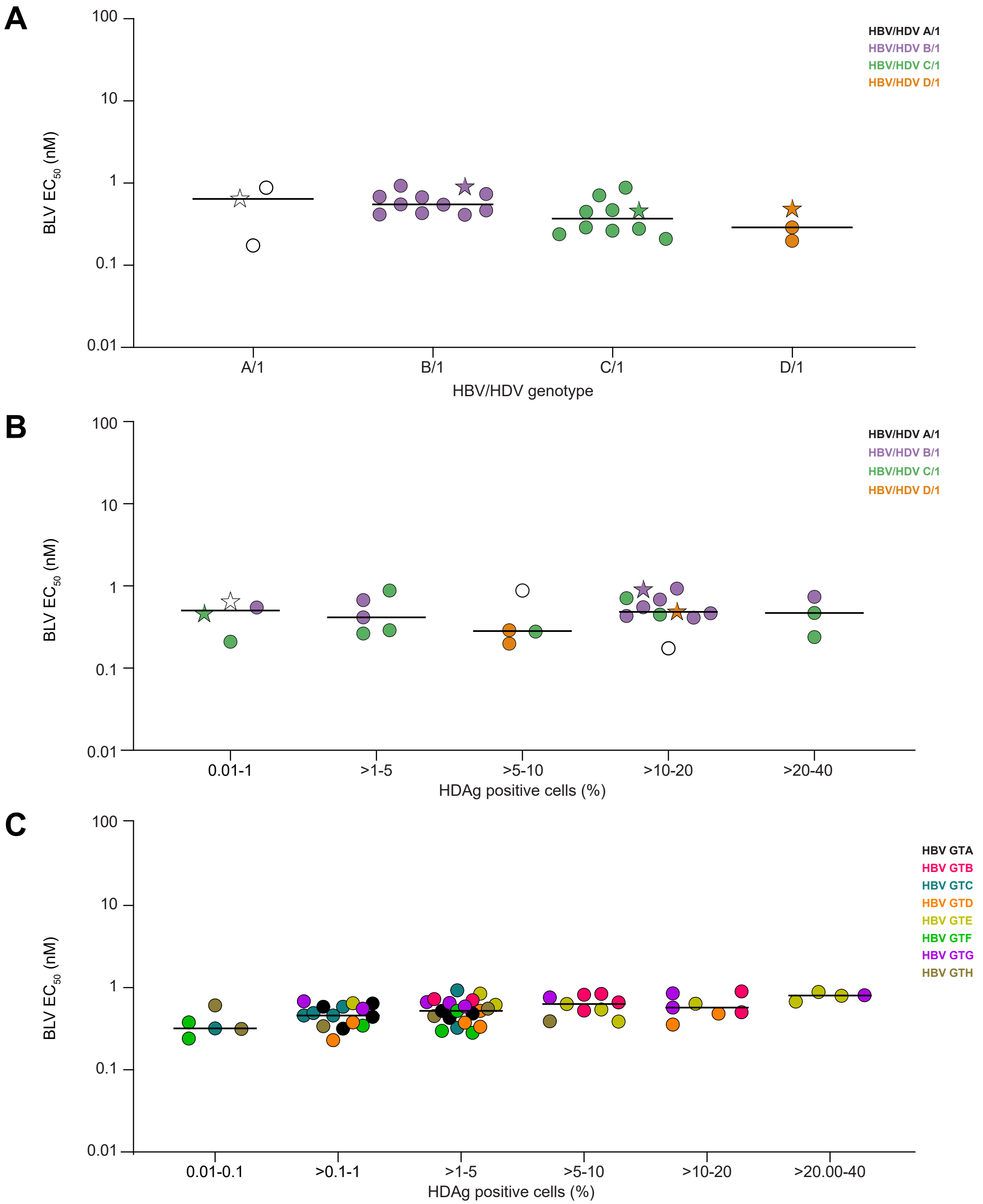

Fig. 3. Relationship between HBV/HDV genotype combination, HDAg positivity, and *in vitro* sensitivity to BLV in laboratory strains. (A) Median BLV EC_{50} values for HDV-1 enveloped with representative sequences of HBV PreS1 GTA–D (stars) or the most common polymorphisms found in clinical samples. Each data point represents the average of two independent experiments. Bars show median values. (B) Median BLV EC_{50} values *vs*. percentage of HDAg-positive cells for HDV-1 enveloped with representative sequences of HBV PreS1 GTA–D (stars) or the most common polymorphisms found in clinical samples. Each data point represents the average of two independent experiments. Bars show median values. (C) Median BLV EC_{50} values as a function of the percentage of HDAg-positive cells for all HBV/HDV combinations tested. Each data point represents the average of at least two independent experiments. Bars show median values. BLV, bulevirtide; GTA–H, genotypes A–H; HDAg, HDV antigen; HDV-1, HDV genotype 1.

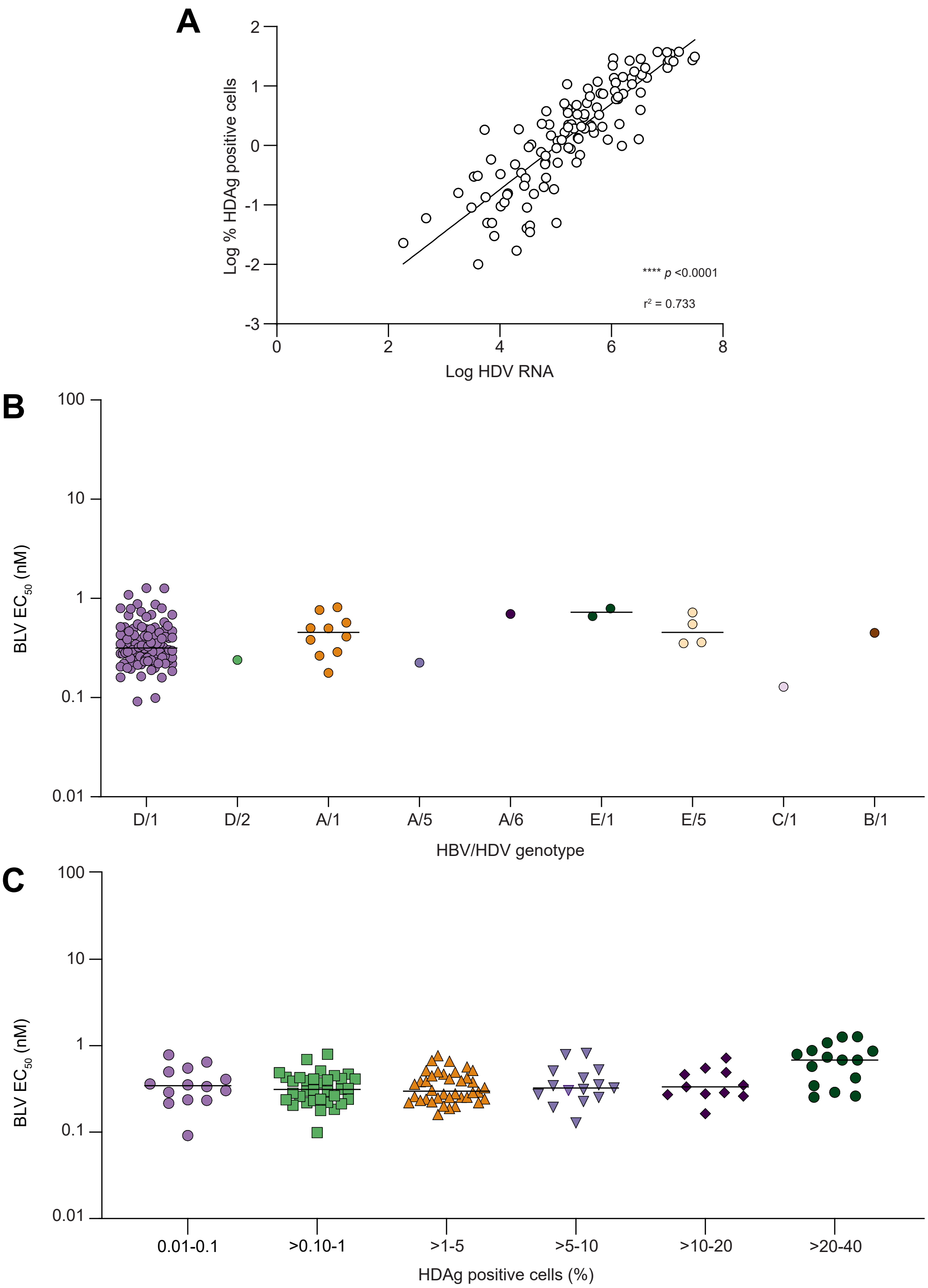

Fig. 4. Effect of HBV/HDV genotype combination, HDAg positivity, and HDV viral load on *in vitro* sensitivity to BLV in clinical samples. (A) Pearson correlation of HDAg-positive cells and HDV RNA (viral load) for 126/128 (98%) clinical samples described in this study (p <0.0001; r^2 = 0.733). (B) Median BLV EC_{50} values plotted by HBV/HDV genotype combination. Each data point represents the average of at least two independent experiments. Bars show median values. (C) Median BLV EC_{50} values stratified by percentage of HDAg positivity for all clinical samples tested. Each data point represents the average of at least two independent experiments. Bars show median values. BLV, bulevirtide; HDAg, HDV antigen.

For all clinical samples tested, the phenotyping assay showed a high correlation between the percentage of HDV-infected cells (percentage of HDAg-positive cells) and the patient's viral load. More than half of the clinical samples tested (72/128, 56%) fell within the 0.1–5% range of HDAg-positive cells when using 2 µl of viral input. Clinical samples that fell in the highest >20% range

Table 3. Antiviral activity of BLV against HDV clinical isolates at baseline.

BLV	HBV/HDV GT								
	D/1 (n = 107)	A/1 (n = 10)	E/5 (n = 4)	C/1 (n = 1)	E/1 (n = 2)	A/6 (n = 1)	B/1 (n = 1)	D/2 (n = 1)	A/5 (n = 1)
Mean EC_{50} ± SD (nM)	0.39 ± 0.22	0.47 ± 0.21	0.50 ± 0.18	0.2 ± 0.08	0.73 ± 0.09	0.70	0.45	0.24	0.23
Median	0.32	0.46	0.46	0.18	0.73	0.70	0.45	0.24	0.23
Range (min–max)	(0.09–1.27)	(0.18–0.64)	(0.35–0.72)	n.a.	(0.66–0.79)	n.a.	n.a.	n.a.	n.a.
5th percentile	0.17	0.18	0.35	0.13	0.66	0.70	0.45	0.24	0.23
95th percentile	0.84	0.82	0.72	0.13	0.79	0.70	0.45	0.24	0.23

Plasma samples from participants at baseline were tested for their sensitivity to BLV in PHHs. Mean, median, range, and the 5th and 95th percentiles are shown.
BLV, bulevirtide; GT, genotype; n.a., not applicable owing to low infectivity; PHH, primary human hepatocyte.

(15/128, 12%) had BLV EC_{50} values that trended slightly higher than those with lower infectivity values although the differences were small (approximately two-fold). Regardless of differences in viral load/infectivity, all clinical samples tested were sensitive to BLV with mean EC_{50} values that ranged from 0.2 to 0.73 nM. Ninety-five percent (121/128) of the plasma samples tested were HDV-1, the most common and diverse HDV genotype. Karimzadeh *et al.*[27] have identified four subgenotypes of HDV-1 with distinct geographic distribution: HDV-1a and HDV-1b in Sub-Saharan Africa; HDV-1c in the Western Pacific region; and HDV-1d, which is more common in Europe and Asia. Our internal phylogenetic analyses have revealed potential new HDV-1 subtypes based on distinct clusters of HDV-1 sequences from the same country.[28] Most of these potential additional subtypes are represented in the clinical samples described here, and despite their sequence divergence (Fig. 5C), they remained highly susceptible to BLV with similar EC_{50} values.

Similar to the clinical samples, the BLV EC_{50} values against various HBV/HDV genotypes for the laboratory strains were within a narrow range from 0.17 to 0.93 nM, with the 95th percentile of EC_{50} values being only two- to three-fold higher than the 5th percentile of EC_{50} values. This <10-fold range in sensitivity to BLV described here is similar to that of sofosbuvir, a pan-genotypic nucleotide inhibitor approved for the treatment of HCV-infected patients, with an EC_{50} range of 0.014 to 0.11 μM for HCV-1 to HCV-6. Other approved HCV inhibitors, such as the NS5A protein-targeting velpatasvir, showed very potent viral inhibition but a much broader range (∼65-fold) of activity depending on the isolate, with EC_{50} values that ranged from 0.002 to 0.13 nM for HCV-1 to HCV-6.[29] This argues for a favourable, broad-spectrum genotypic activity profile for BLV that resembles that of nucleotide inhibitors already available in the clinic for other hepatotropic viruses.

The analysis of the sensitivity of laboratory strains and clinical plasma samples to BLV described here was based on an infection assay using PHH. The percentage of HDAg-positive cells in the assay could be used as a proxy for the *infectivity* of the viruses tested. For laboratory strains, viruses were harvested on Day 8 after cotransfection and then used to infect PHH for 5 days using a constant viral inoculum. Therefore, the assay could reflect the kinetics of infectious HDV viral production for the various HBV/HDV genotype combinations. The infectivity of HDV genotypes, based on median infectivity values of all HBV/HDV combinations, ranked as follows: HDV-3 >HDV-6 ≈ HDV-8 >HDV-5 >HDV-1 >HDV-7 >HDV-2 >HDV-4 (Table S3). For HBV, the infectivity of the eight genotypes ranked as follows: GTE >GTB >GTG >GTD >GTA ≈ GTF >GTC >GTH (Table S3). Overall, most HDV genotypes, namely, HDV-3, HDV-6, HDV-8, HDV-5, and HDV-1, resulted in median infectivity values higher than 2%, whereas other genotypes resulted in very low infectivity, particularly HDV-4, with a median value of 0.05%. For HDV-4, combinations C/4 and D/4 had infectivity values <0.05%, whereas combinations A/4, F/4, and H/4 resulted in no detectable infectivity in the assay. Interestingly, for HDV-6, no infectivity was detected for combinations F/6 and H/6; however, all other combinations resulted in infectivity values that allowed for phenotyping against BLV. Consistent with other reports, our data show that the kinetics of formation of infectious HDV viral particles may vary depending on the particular HBV/HDV genotype combination.[16] In fact, not all HDV genotypes were enveloped with the same efficiency by L-HBsAg envelopes from different HBV genotypes. Selective preference for envelopes of certain HBV genotypes in HDV genomic assembly has been described before. It was hypothesised that differences of assembly kinetics and/or *de novo* cell entry of some of the combinations may explain this phenomenon.[16] It is likely that in addition to sensitivity to BLV, the virological response in patients will depend also on other factors, such as cell turnover, RNA decay, and the immune response. Although the sensitivity of all HDV/HBV genotypes to BLV in this single-round *in vitro* infection assay is very similar, the virological responses in patients infected with different genotypes and treated with BLV will warrant further investigation.[30,31]

This is the first report of BLV's *in vitro* activity on a wide and representative collection of samples that include genotype combinations with very low infectivity that had hampered previous attempts at phenotyping. This was achieved through the establishment of a sensitive infectivity assay using PHHs. These promising results, with a large number of samples all susceptible to *in vitro* BLV inhibition, will have to be confirmed *in vivo* in patients with more prevalent HBV/HDV genotype combinations in circulation and rare HDV genotypes. The natural occurrence of HBV/HDV genotypes has been described in some studies: in China, HDV-1 was mainly associated with HBV GTC, although HDV-2 was also reported.[32] In Taiwan, HDV-4 was the prevalent genotype (72%) circulating among injection drug users, whereas HDV-2 was predominant (73%) in the non-injection drug use populations.[33] These HBV/HDV genotype combinations prevalent in China and Taiwan show similar *in vitro* BLV sensitivity compared to any other HBV/HDV genotype. However, clinical samples with these genotype combinations will need to be further investigated.

In summary, this study presents data on the potent activity of BLV on the most diverse collection of clinical samples from patients with CHD reported to date. Although the majority of the

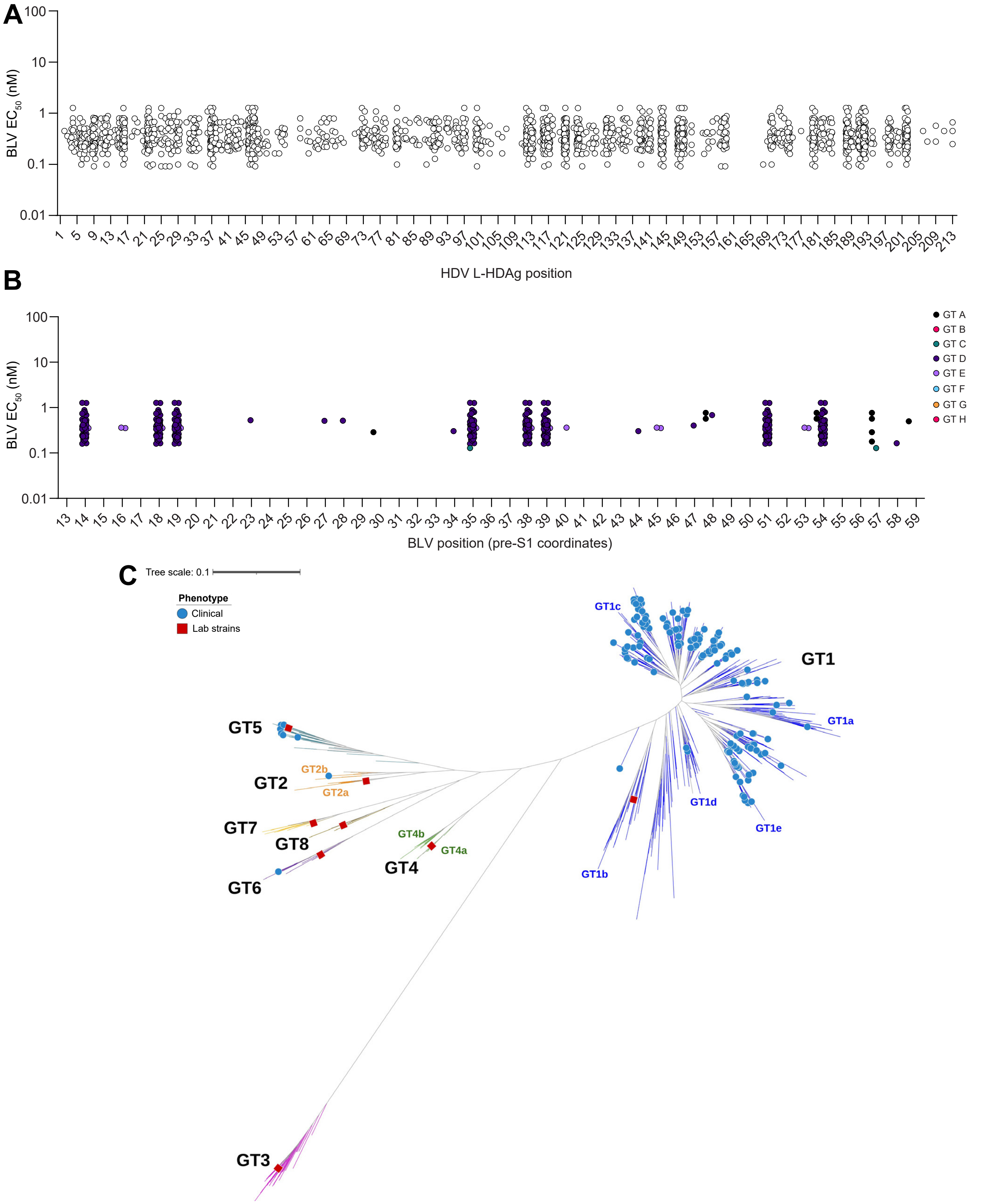

Fig. 5. Effect of HDAg and HBV PreS1 polymorphisms on *in vitro* sensitivity to BLV. (A) Median BLV EC_{50} values plotted against polymorphic positions in HADg from HDV-1. Each data point denotes a sample (clinical or laboratory strains) that contains a variant different from that of a consensus HDV-1 sequence derived from one representative sequence per HDV-1 subtype out of 10 reference sequences. (B) Median BLV EC_{50} values plotted against polymorphic positions of the BLV region of PreS1. Each data point represents a sample with a variant amino acid position different from the BLV sequence. (C) Phylogenetic tree of HDV genome highlighting all *in vitro* phenotyped laboratory strains (red squares) and clinical (blue circles) for their sensitivity to BLV. BLV, bulevirtide; GTA–H, genotypes A–H; HDAg, HDV antigen; HDV-1 to HDV-8, HDV genotypes 1–8.

samples were HDV-1, some other less frequently found genotypes are described, such as HDV-2, -5, and -6. Overall, all isolates and laboratory strains displayed similar *in vitro* sensitivity to BLV, supporting its use as a novel therapy for patients with CHD, regardless of the particular combination of HBV/HDV genotypes they are infected with.

Abbreviations

BLV, bulevirtide; CHD, chronic hepatitis D; GTA–H, genotypes A–H; HDAg, HDV antigen; HDV-1–8, HDV genotypes 1–8; NCTP, sodium taurocholate cotransporting polypeptide; PHH, primary human hepatocyte.

Financial support

Funding was received from Gilead Sciences.

Conflicts of interest

DA and JH report no conflicts of interest. RMat, YL, SX, SC, RM, SM, TAe, BH, TY, LM, DH, AS, RMar, DM, CR, EM, CM, and HM are employees and stockholders of Gilead Sciences. Tas and PL are consultants for Gilead Sciences. SU is a co-applicant and co-inventor on patents protecting Hepcludex (bulevirtide/Hepcludex) and consultant for Gilead Sciences. Please refer to the accompanying ICMJE disclosure forms for further details.

Authors' contributions

Writing of the manuscript: Rmat, HM. Data analysis: Rmat, HM. Interpretation of the data: Rmat, HM, SX, AS, TY, YL, LM, BH, DH, Rmar, SM, CR, CM, Tae, SC, DM, EM, HM, JH, SU, Tas, DA, PL. Experimental supervision: SX, AS, TY, YL, LM, BH, DH, Rmar, SM, CR, CM, Tae, SC, DM, EM, HM. Manuscript review: JH, SU, Tas, DA, PL. Sample supply: Tas, DA, PL. Manuscript drafting and review: all authors.

Data availability statement

The data of this study are available upon reasonable request.

Supplementary data

Supplementary data to this article can be found online at https://doi.org/10.1016/j.jhepr.2023.100893.

References

Author names in bold designate shared co-first authorship

[1] Sagnelli C, Sagnelli E, Russo A, Pisaturo M, Occhiello L, Coppola N. HBV/HDV co-infection: epidemiological and clinical changes, recent knowledge and future challenges. Life (Basel) 2021;11:169.
[2] Romeo R, Del Ninno E, Rumi M, Russo A, Sangiovanni A, de Franchis R, et al. A 28-year study of the course of hepatitis Δ infection: a risk factor for cirrhosis and hepatocellular carcinoma. Gastroenterology 2009;136:1629–1638.
[3] Stockdale AJ, Kreuels B, Henrion MYR, Giorgi E, Kyomuhangi I, de Martel C, et al. The global prevalence of hepatitis D virus infection: systematic review and meta-analysis. J Hepatol 2020;73:523–532.
[4] Miao Z, Zhang S, Ou X, Li S, Ma Z, Wang W, et al. Estimating the global prevalence, disease progression, and clinical outcome of hepatitis Delta Virus Infection. J Infect Dis 2020;221:1677–1687.
[5] Papatheodoridi M, Papatheodoridis GV. Is hepatitis delta underestimated? Liver Int 2021;41(Suppl. 1):38–44.
[6] **Urban S**, **Neumann-Haefelin C**, **Lampertico P**. Hepatitis D virus in 2021: virology, immunology and new treatment approaches for a difficult-to-treat disease. Gut 2021;70:1782–1794.
[7] Khalfi P, Kennedy PT, Majzoub K, Asselah T. Hepatitis D virus: improving virological knowledge to develop new treatments. Antivir Res 2023;209:105461.
[8] Lempp FA, Ni Y, Urban S. Hepatitis delta virus: insights into a peculiar pathogen and novel treatment options. Nat Rev Gastroenterol Hepatol 2016;13:580–589.
[9] Rizzetto M. Hepatitis D: thirty years after. J Hepatol 2009;50:1043–1050.
[10] Sureau C, Guerra B, Lanford RE. Role of the large hepatitis B virus envelope protein in infectivity of the hepatitis delta virion. J Virol 1993;67:366–372.
[11] Taylor JM. Hepatitis D virus replication. Cold Spring Harb Perspect Med 2015;5:a021568.
[12] Wedemeyer H, Manns MP. Epidemiology, pathogenesis and management of hepatitis D: update and challenges ahead. Nat Rev Gastroenterol Hepatol 2010;7:31–40.
[13] Tu T, Urban S. Virus entry and its inhibition to prevent and treat hepatitis B and hepatitis D virus infections. Curr Opin Virol 2018;30:68–79.
[14] Sunbul M. Hepatitis B virus genotypes: global distribution and clinical importance. World J Gastroenterol 2014;20:5427–5434.
[15] Wang PY, Yang X, Guo L, Wang YW, Zhang WL, Sun YX, et al. Establishment of a human cell line with a surface display system for screening and optimizing Na^+-taurocholate cotransporting polypeptide-binding peptides. Front Microbiol 2022;13:920280.
[16] Wang W, Lempp FA, Schlund F, Walter L, Decker CC, Zhang Z, et al. Assembly and infection efficacy of hepatitis B virus surface protein exchanges in 8 hepatitis D virus genotype isolates. J Hepatol 2021;75:311–323.
[17] Ni Y, Lempp FA, Mehrle S, Nkongolo S, Kaufman C, Fälth M, et al. Hepatitis B and D viruses exploit sodium taurocholate co-transporting polypeptide for species-specific entry into hepatocytes. Gastroenterology 2014;146:1070–1083.
[18] Yan H, Zhong G, Xu G, He W, Jing Z, Gao Z, et al. Sodium taurocholate cotransporting polypeptide is a functional receptor for human hepatitis B and D virus. eLife 2012;1:e00049.
[19] Degasperi E, Anolli MP, Lampertico P. Bulevirtide for patients with compensated chronic hepatitis delta: a review. Liver Int 2023;(Suppl. 1):80–86.
[20] Wedemeyer H, Schöneweis K, Bogomolov P, Blank A, Voronkova N, Stepanova T, et al. Safety and efficacy of bulevirtide in combination with tenofovir disoproxil fumarate in patients with hepatitis B virus and hepatitis D virus coinfection (MYR202): a multicentre, randomised, parallel-group, open-label, phase 2 trial. Lancet Infect Dis 2023;23:117–129.
[21] Herta T, Hahn M, Maier M, Fischer J, Niemeyer J, Honemann M, et al. Efficacy and safety of bulevirtide plus tenofovir disoproxil fumarate in real-world patients with chronic hepatitis B and D co-infection. Pathogens 2022;11:517.
[22] Loglio A, Ferenci P, Uceda Renteria SC, Tham CYL, Scholtes C, Holzmann H, et al. Safety and effectiveness of up to 3 years' bulevirtide monotherapy in patients with HDV-related cirrhosis. J Hepatol 2022;76:464–469.
[23] Asselah T, Loureiro D, Le Gal F, Narguet S, Brichler S, Bouton V, et al. Early virological response in six patients with hepatitis D virus infection and compensated cirrhosis treated with Bulevirtide in real-life. Liver Int 2021;41:1509–1517.
[24] Lampertico P, Roulot D, Wedemeyer H. Bulevirtide with or without pegIFNα for patients with compensated chronic hepatitis delta: from clinical trials to real-world studies. J Hepatol 2022;77:1422–1430.
[25] Parallel Sessions. J Hepatol 2021;75(Suppl. 2):S205–S293.
[26] Oral Presentations. J Hepatol 2022;77(Suppl. 1):S1–S18.
[27] Karimzadeh H, Usman Z, Frishman D, Roggendorf M. Genetic diversity of hepatitis D virus genotype-1 in Europe allows classification into subtypes. J Viral Hepat 2019;26:900–910.
[28] Manhas S, Xu S, Chang S, Aeschbacher T, Mateo R, Martin R, et al. HDV full genome sequencing and sensitive HBV genotyping from a large cohort of HBV/HDV co-infected patients. EASL 2023 Poster.
[29] Cory TJ, Mu Y, Gong Y, Kodidela S, Kumar S. Sofosbuvir + velpatasvir + voxilaprevir for the treatment of hepatitis C infection. Expert Opin Pharmacother 2018;19:749–757.
[30] Sureau C, Negro F. The hepatitis delta virus: replication and pathogenesis. J Hepatol 2016;64:S102–S116.
[31] Usman Z, Velkov S, Protzer U, Roggendorf M, Frishman D, Karimzadeh H. HDVdb: a comprehensive hepatitis D virus database. Viruses 2020;12:538.
[32] Wu S, Zhang Y, Tang Y, Yao T, Lv M, Tang Z, et al. Molecular epidemiology and clinical characteristics of hepatitis delta virus (HDV) infected patients with elevated transaminases in Shanghai, China. BMC Infect Dis 2020;20:565.
[33] Lin HH, Lee SS, Yu ML, Chang TT, Su CW, Hu BS, et al. Changing hepatitis D virus epidemiology in a hepatitis B virus endemic area with a national vaccination program. Hepatology 2015;61:1870–1879.

Journal of Hepatology, Volume *5*

Supplemental information

Broad-spectrum activity of bulevirtide against clinical isolates of HDV and recombinant pan-genotypic combinations of HBV/HDV

Roberto Mateo, Simin Xu, Alex Shornikov, Tahmineh Yazdi, Yang Liu, Lindsey May, Bin Han, Dong Han, Ross Martin, Savrina Manhas, Christopher Richards, Caleb Marceau, Thomas Aeschbacher, Silvia Chang, Dmitry Manuilov, Julius Hollnberger, Stephan Urban, Tarik Asselah, Dzhamal Abdurakhmanov, Pietro Lampertico, Evguenia Maiorova, and Hongmei Mo

Broad-spectrum activity of bulevirtide against clinical isolates of HDV and recombinant pan-genotypic combinations of HBV/HDV

Roberto Mateo, Simin Xu, Alex Shornikov, Tahmineh Yazdi, Yang Liu, Lindsey May, Bin Han, Dong Han, Ross Martin, Savrina Manhas, Christopher Richards, Caleb Marceau, Thomas Aeschbacher, Silvia Chang, Dmitry Manuilov, Julius Hollnberger, Stephan Urban, Tarik Asselah, Dzhamal Abdurakhmanov, Pietro Lampertico, Evguenia Maiorova, Hongmei Mo

Table of contents

Table S1 2

Table S2 3

Table S3 4

Table S4 6

Table S1. Percentage of HDAg-positive PHH cells after infection with HDV-1-8 enveloped with L-HBsAg from HBV GTA-H

	BLV EC_{50} (nM)									
HBV GT	HDV-1	HDV-2	HDV-3	HDV-4	HDV-5	HDV-6	HDV-7	HDV-8	HBV mean	HBV median
GTA	0.21 ± 0.03	0.17 ± 0.04	0.66 ± 0.33	0#	2.55 ± 0.07	4.19 ± 0.30	0.95 ± 0.38	3.75 ± 0.07	1.56	0.81
GTB	18 ± 5.63	8.27 ± 0.29	10.77 ± 3.60	2.27 ± 0.48	6.91 ± 0.21	9.84 ± 0.44	5.54 ± 0.25	4.38 ± 0.06	8.36	7.59
GTC	0.77 ± 0.14	0.39 ± 0.07	0.08 ± 0.04	0.02 ± 0.01	2.55 ± 0.28	0.54 ± 0.17	0.21 ± 0.04	3.20 ± 0.09	0.97	0.47
GTD	10.1 ± 5.54	0.53 ± 0.03	12.40 ± 2.27	0.05 ± 0.0	1.45 ± 0.27	2.07 ± 0.35	0.30 ± 0.03	1.94 ± 0.10	3.60	1.70
GTE	2.76 ± 0.27	6.60 ± 0.57	6.96 ± 1.60	0.47 ± 0.01	34..42[a]	24.77[a]	25.14 ± 3.68	11.36 ± 1.25	14.06	9.16
GTF	1.19 ± 0.43	0.02 ± 0.01	1.96 ± 0.33	0	0.38 ± 0.16	0	0.08 ± 0.02	4.09 ± 0.23	1.29	0.79
GTG	13.3 ± 4.68	0.86 ± 0.11	6.40 ± 0.41	0.36 ± 0.14	24.94[a]	3.88 ± 1.16	12.85 ± 4.53	4.00 ± 0.08	8.32	5.20
GTH	1.57 ± 0.47	0.08 ± 0.04	5.40 ± 0.40	0	0.52 ± 0.13	0	0.03 ± 0.01	3.53 ± 0.03	1.39	0.30
HDV mean	6.09	2.12	5.58	0.45	9.21	6.47	5.64	4.53		
HDV median	2.16	0.46	5.90	0.05	2.55	3.88	0.63	3.88		

[a] Assay used 40 µl of input viral inoculum (N=1)

Table S2. Activity of BLV against HDV genotype 1 enveloped with representative sequences of preS1 region for HBV genotypes A-H

Position[a]	Average EC_{50} (nM)[c]	15 20 25 30 35 40 45 50 55
BLV	-	GT N LSVP N PLGF F PDHQ L DPAF G ANSN N PDWD F NPNK D HWPE A NKVG
GTA pLX304-HB2.7-gtA	0.65	. . . . V . . . . I . . . . . A . . Q . .
GTB pLX304-HB2.7-gtB	0.90	. . . . K . . . E . . . . . L . . Y . . N . . D . . . . .
GTC pLX304-HB2.7-gtC	0.46	. . . . Q . .
GTD pLX304-HB2.7-gtD	0.49	. Q . . . T S . . . . R . . T A . . . . T . . D . . . . .
GTE pLX304-HB2.7-gtE	0.85	. K . H . T T . . . . R . . T R . . . . . H . . . . T . . . . . .
GTF pLX304-HB2.7-gtF	0.28	. Q . . . . L . R . . . S S . . . . . . T . . . S . . M . . . . .
GTG pLX304-HB2.7-gtG	0.57	. K . . . T S . . . . . L . . . . R . . T . . . . K . . P . . . .
GTH pLX304-HB2.7-gtH	0.45	. Q . . . . L . R . . . S S . . . . . . T . . . N . . M . . . . .

[a] Numbering corresponds to positions of genotype A X02763
[b] BLV sequence was used as a reference for each position
[c] Average of two replicate values is shown (N=2)

Table S3. Sequence alignment of BLV sequence with the most common clinical isolates of HBV genotypes A-D

Position	Average EC50 (nM)[a]	15 20 25 30 35 40 45 50 55
BLV Sequence	-	GTN LSVPN PLGFF PDHQL DPAFG ANSNN PDWDF NPNKD HWPEA NKVG
GTA Consensus	0.65[b]	... .I.. ...A. .Q..
HBVA.1-HDV1	0.17	... .A... .... .L..R ....V.. -..Q. .Q..
HBVA.2-HDV1	0.88	... .I.. ...Q. .Q..
GTB Consensus	0.90[b]	... .K ...E. ....L ..H.. N..D. ....
HBVB.1-HDV1	0.47	... .A... ....LR ...E. ....L ..H.. N..D. ....
HBVB.2-HDV1	0.74	... .L ....K ...D. ....L ..H.. T..DS ....
HBVB.3-HDV1	0.55	... .L ....K ...D. ....L ..H.. N..D. ....
HBVB.4-HDV1	0.42	... .K ...D. ....L ..H.. Y..DS ....
HBVB.5-HDV1	0.41	... .K ...E. ..... .QH.. N..D. ....
HBVB.6-HDV1	0.93	... .K ...E. ....L ..Y.. N..D. ....
HBVB.7-HDV1	0.67	... .K ...E. ....L ..Y.. N..D. ....
HBVB.8-HDV1	n/a	... .K ...E. ....L ..H.. T..D. ....
HBVB.9-HDV1	0.68	... .K ...E. ....L ..Y.. N..D. ....
HBVB.10-HDV1	0.43	... .K ...E. ....L ..H.. N..N. ....
GTC Consensus	0.46[a]	... . .Q..
HBVC.1-HDV1	0.47	... .A... .... .L..R .... P.... .Q..
HBVC.2-HDV1	0.21	... .A... .... .L..R .... P.... HQ..
HBVC.3-HDV1	0.71	... .L .... .Q..
HBVC.4-HDV1	0.24	... .A... .... .L..R .... P.... .Q..
HBVC.5-HDV1	0.45	... . .G... ....R .... Q..A. .Q..
HBVC.6-HDV1	0.28	... . .G... ....L ..... Q..A. HQ..
HBVC.7-HDV1	0.88	... .L .... P.... WQ..
HBVC.8-HDV1	0.29	... . .G... .... Q..A. .Q..
HBVC.9-HDV1	n/a	... . Q.... .Q..

**HBVC.10-HDV1**	0.26	. . . . G. . . . . Q. . A. . Q. .

**GTD Consensus**	0.49[a]	. Q. . . TS. . . . . R . . TA. . . . . T. . D. . . . .
**HBVD.1-HDV1**	0.29	. Q. . . TS. . . . . K . . TA. . . . . P. . D. . . . .
**HBVD.2-HDV1**	0.2	. Q. . . TS. . . . . R . . TA. . . . . T. . N. . . . .

[a] Average of two replicate values is shown (N=2)
[b] EC_{50} for representative sequences for HBV genotypes A-D were measured as described in Table 1
n/a: EC_{50} values not available

Table S4. Subject clinical, virological and biochemical information for baseline plasma samples from participants described in this study

Subject ID	Country[a]	HBV GT	HDV GT	Log10 HDV RNA [IU/mL]	HBV DNA [IU/mL]	Log10 HBsAg [IU/mL]	EC_{50}	ALT [U/mL]
1	RUS	D	1	4.81	<10	3.18	0.37	ND
2	RUS	D	1	4.74	<10	3.52	0.22	ND
3	RUS	D	1	7.01	22	3.08	0.69	ND
4	RUS	D	1	5.21	15	3.92	0.26	ND
5	RUS	D	1	6.53	26	3.88	0.25	ND
6	RUS	D	1	6.64	10	4.08	0.16	ND
7	RUS	A	1	6.52	522	3.73	0.57	ND
8	RUS	D	1	7.01	60	4.23	0.68	ND
9	RUS	D	1	6.21	22	3.40	0.19	ND
10	RUS	D	1	4.01	<10	2.85	0.23	439
11	FRA	A	1	5.23	20	3.96	0.77	ND
12	FRA	E	5	5.85	768	4.18	0.35	ND
13	FRA	A	6	4.13	16	3.45	0.70	59
14	FRA	E	5	6.37	ND	3.79	0.72	76
15	RUS	D	1	3.75	14	3.91	0.40	81
16	RUS	D	1	4.49	71	4.00	0.41	113
17	RUS	D	1	5.04	19	3.76	0.24	302
18	RUS	D	1	6.55	13	4.48	0.27	104
19	RUS	D	1	7.46	11	4.15	1.27	94
20	RUS	D	1	5.69	12	3.76	0.67	42
21	RUS	D	1	4.56	12	3.83	0.47	45
22	RUS	D	1	7.06	172	4.18	0.88	70
23	RUS	D	1	4.92	<10	2.49	0.29	335
24	RUS	D	1	4.15	<10	3.91	0.47	49
25	RUS	D	1	6.52	28	4.23	0.27	95
26	RUS	D	1	3.78	66	3.76	0.24	ND
27	RUS	D	1	6.03	93	ND	0.87	ND
28	RUS	D	1	4.83	21	3.77	0.50	82
29	RUS	D	1	6.49	16	2.70	0.31	133
30	RUS	D	1	4.83	10	3.66	0.43	36
31	RUS	D	1	5.56	185	3.32	0.42	82
32	RUS	D	1	4.97	43333	3.90	0.39	193
33	RUS	D	1	4.61	<10	3.75	0.51	91
34	RUS	D	1	7.49	22	4.30	0.43	285
35	RUS	D	1	4.79	12	3.95	0.32	40
36	RUS	D	1	3.86	10	2.54	0.34	64
37	RUS	D	1	5.27	<10	3.57	0.80	39
38	RUS	D	1	3.90	57	3.69	0.22	212
39	RUS	D	1	5.58	3318	4.46	0.32	123

40	RUS	D	1	5.50	39	4.18	0.26	145
41	RUS	D	1	6.05	148	4.43	0.28	273
42	RUS	D	1	3.84	11	3.04	0.49	273
43	RUS	D	1	5.40	<10	4.11	0.20	107
44	RUS	D	1	4.01	<10	4.11	0.42	ND
45	RUS	D	1	5.38	317	3.36	0.39	ND
46	RUS	D	1	5.28	13	3.30	0.43	73
47	RUS	D	1	5.16	<10	3.94	0.25	125
48	RUS	D	1	6.08	418	4.28	0.35	54
49	RUS	D	1	7.04	3343101	3.99	1.27	210
50	RUS	D	1	4.39	26	3.58	0.30	38
51	RUS	D	1	6.06	125	4.15	0.31	141
52	RUS	D	1	6.12	101	4.20	0.23	101
53	RUS	D	1	5.63	17	3.91	0.28	97
54	RUS	D	1	6.19	152	3.59	0.45	130
55	RUS	D	1	2.27	6840	3.61	0.79	51
56	RUS	D	1	3.54	<10	3.69	0.26	84
57	RUS	D	1	7.11	<10	3.65	0.58	88
58	RUS	D	1	5.22	11	3.65	0.24	103
59	RUS	D	1	6.08	454	3.73	0.25	54
60	RUS	D	1	2.68	81	3.48	0.55	64
61	RUS	D	1	5.02	<10	4.23	0.09	101
62	RUS	D	1	5.16	<10	4.00	0.53	57
63	RUS	D	1	5.75	<10	3.92	0.29	80
64	RUS	D	1	6.12	<10	3.69	0.34	52
65	RUS	D	1	6.36	274	3.65	0.49	184
66	RUS	D	1	6.61	22	4.30	0.29	ND
67	RUS	D	1	5.04	14	3.89	0.28	25
68	RUS	D	1	4.46	12	3.59	0.31	40
69	RUS	A	1	6.14	<10	3.26	0.38	239
70	RUS	D	1	5.81	737	3.93	0.51	ND
71	RUS	D	1	5.62	16	3.99	0.30	ND
72	RUS	D	1	4.89	<10	2.74	0.24	136
73	RUS	D	1	4.81	2152113	4.36	0.41	ND
74	RUS	D	1	5.52	<10	3.54	0.25	76
75	RUS	D	1	4.08	56	3.82	0.40	43
76	RUS	D	1	4.34	16	3.67	0.25	ND
77	RUS	D	1	5.11	54	4.00	0.22	ND
78	RUS	D	1	5.23	<10	4.43	0.18	77
79	RUS	D	1	4.82	17	3.64	0.28	ND
80	DEU	A	1	5.65	20	3.34	0.50	120
81	DEU	D	1	4.52	<10	3.23	0.32	55
82	DEU	D	1	6.02	23	4.26	0.26	74
83	DEU	D	1	5.52	<10	3.87	0.16	77
84	DEU	D	1	4.44	518	4.28	0.21	114
85	DEU	D	1	5.44	<10	2.92	0.20	254
86	DEU	D	1	5.39	23	4.08	0.19	74
87	DEU	D	1	4.49	<10	3.95	0.16	74

88	DEU	D	1	5.74	<10	4.15	0.20	197
89	DEU	D	1	6.83	<10	4.04	0.74	63
90	DEU	D	1	3.60	11	3.18	0.24	124
91	DEU	D	1	5.01	21	3.95	0.23	113
92	DEU	D	1	3.49	<10	ND	0.30	ND
93	DEU	D	1	6.32	166	ND	0.35	ND
94	DEU	D	1	4.30	<10	2.89	0.65	289
95	DEU	D	1	5.78	42	4.20	0.52	247
96	DEU	E	5	4.54	27	3.83	0.36	ND
97	DEU	D	1	4.54	<10	2.32	0.29	128
98	DEU	D	1	5.33	<10	2.76	0.25	266
99	ITA	D	1	3.61	170	2.34	0.35	63
100	ITA	D	1	5.22	23	3.56	0.22	57
101	ITA	D	1	5.22	18	3.30	0.39	90
102	ITA	D	1	7.00	13	3.36	0.80	102
103	ITA	D	1	5.84	<10	3.80	0.23	65
104	ITA	D	1	5.38	10	4.00	0.43	59
105	ITA	D	1	6.41	14	4.11	0.33	131
106	ITA	D	1	5.41	<10	3.18	0.39	191
107	ITA	D	1	5.46	12	4.04	0.52	36
108	ITA	D	1	7.21	35	4.20	1.08	245
109	ITA	D	1	6.21	<10	3.91	0.47	146
110	ITA	D	1	3.73	<10	3.51	0.33	116
111	ITA	D	1	3.26	<10	3.79	0.22	76
112	ITA	D	1	5.93	<10	3.69	0.36	161
113	ITA	D	1	4.28	<10	3.83	0.30	39
114	ITA	D	1	4.75	61	3.65	0.41	ND
115	SWE	A	1	4.48	42	3.49	0.50	ND
116	CIV	A	1	ND	ND	ND	0.29	144
117	CIV	A	5	ND	<10	ND	0.23	36
118	CIV	E	5	ND	490	ND	0.55	42
119	MDA	A	1	ND	<10	ND	0.18	206
120	RUS	D	1	ND	<10	ND	0.10	19
121	CMR	E	1	ND	15100	ND	0.79	72
122	ROU	A	1	ND	ND	ND	0.41	226
123	RUS	D	2	ND	ND	ND	0.24	ND
124	RUS	A	1	ND	ND	ND	0.27	ND
125	FRA	C	1	ND	<20	ND	0.13	60
126	FRA	E	1	ND	27	ND	0.66	35
127	ITA	B	1	ND	ND	ND	0.45	ND
128	ITA	A	1	ND	ND	ND	0.82	ND

[a] RUS = Russia; FRA = France; DEU = Germany; ITA = Italy; SWE = Sweden; CIV = Cote D'Ivoire; MDA = Moldova; CMR = Cameroon; ROU = Romania
